# Supplementary material for: CLEC5A is a critical receptor in innate immunity against Listeria infection
Source: Nat Commun. 2017 Aug 21;8:299. doi: 10.1038/s41467-017-00356-3 (PMC5563510; doi:10.1038/s41467-017-00356-3)
Supplement: Supplementary file 1 — Supplementary information [file 41467_2017_356_MOESM1_ESM.pdf]

Title of file for HTML: Supplementary Information
Description: Supplementary figures, supplementary tables

Title of file for HTML: Peer Review File
Description:

Supplementary Figure 1

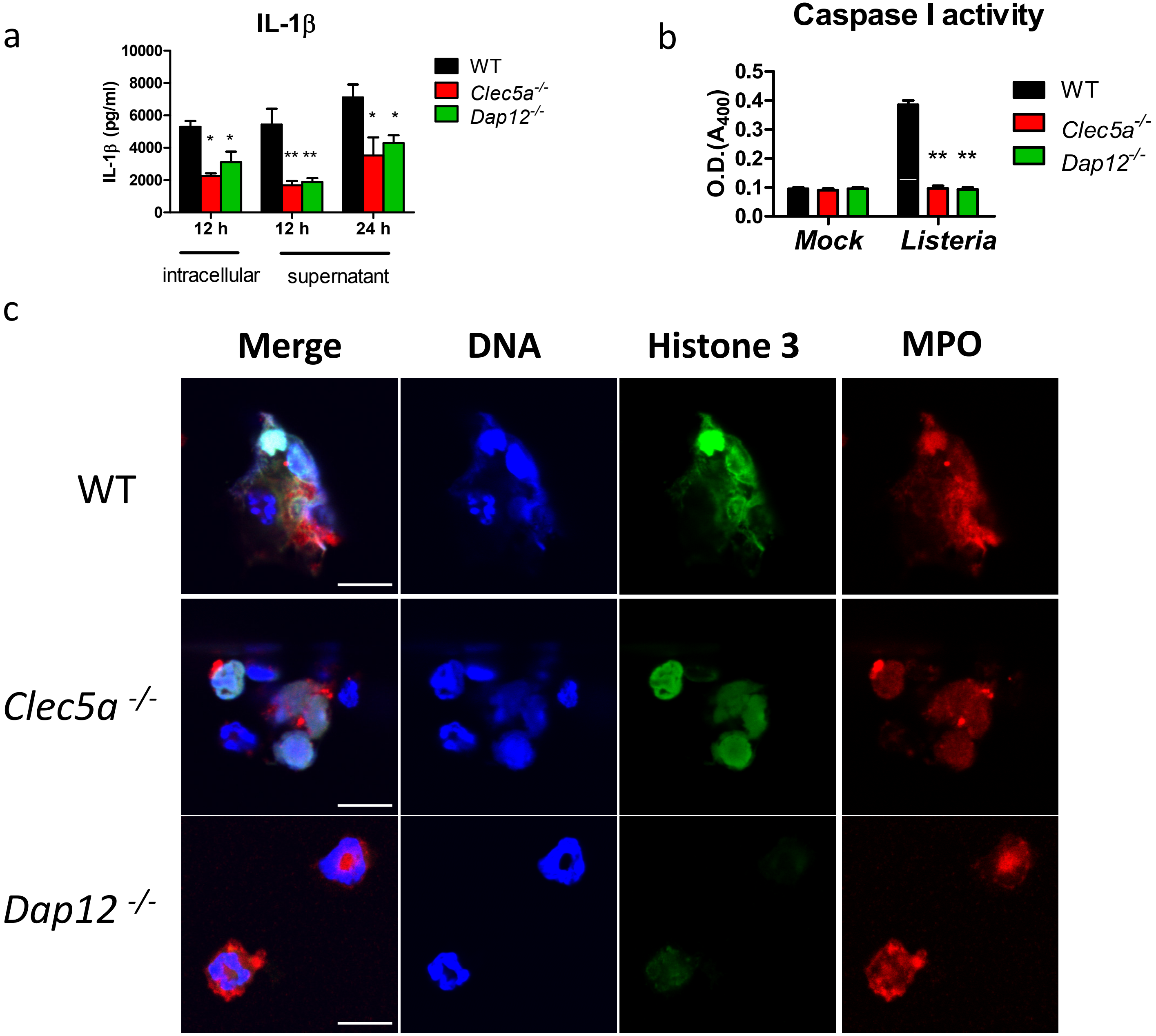

Supplementary Figure 1. DAP12 is critical for IL-1β expression in macrophages and NET formation in response to *L. monocytogenes.*

(**a**) Mouse macrophages were incubated with live *L. monocytogenes* and supernatants were harvested. IL-1β secretion was measured by ELISA. (**b**) Caspase-1 activity in mouse macrophages (6 h post *Listeria* infection) was presented as absorbance at 405 nm (A_{405}). Data were collected and expressed as mean ± s.e.m. from at least three independent experiments. One-way ANOVA was performed. *P<0.05, ** P<0.01 for wild type (WT) versus knockout mice. (**c**) Mouse neutrophils were incubated with *Listeria* (MOI 10) for 90 min, cells were fixed and then stained with Hoechst 33342 (blue), anti-MPO (red) and anti-histone (green) polyclone antibodies. Scale bar, 20 μm.

Supplementary Figure 2

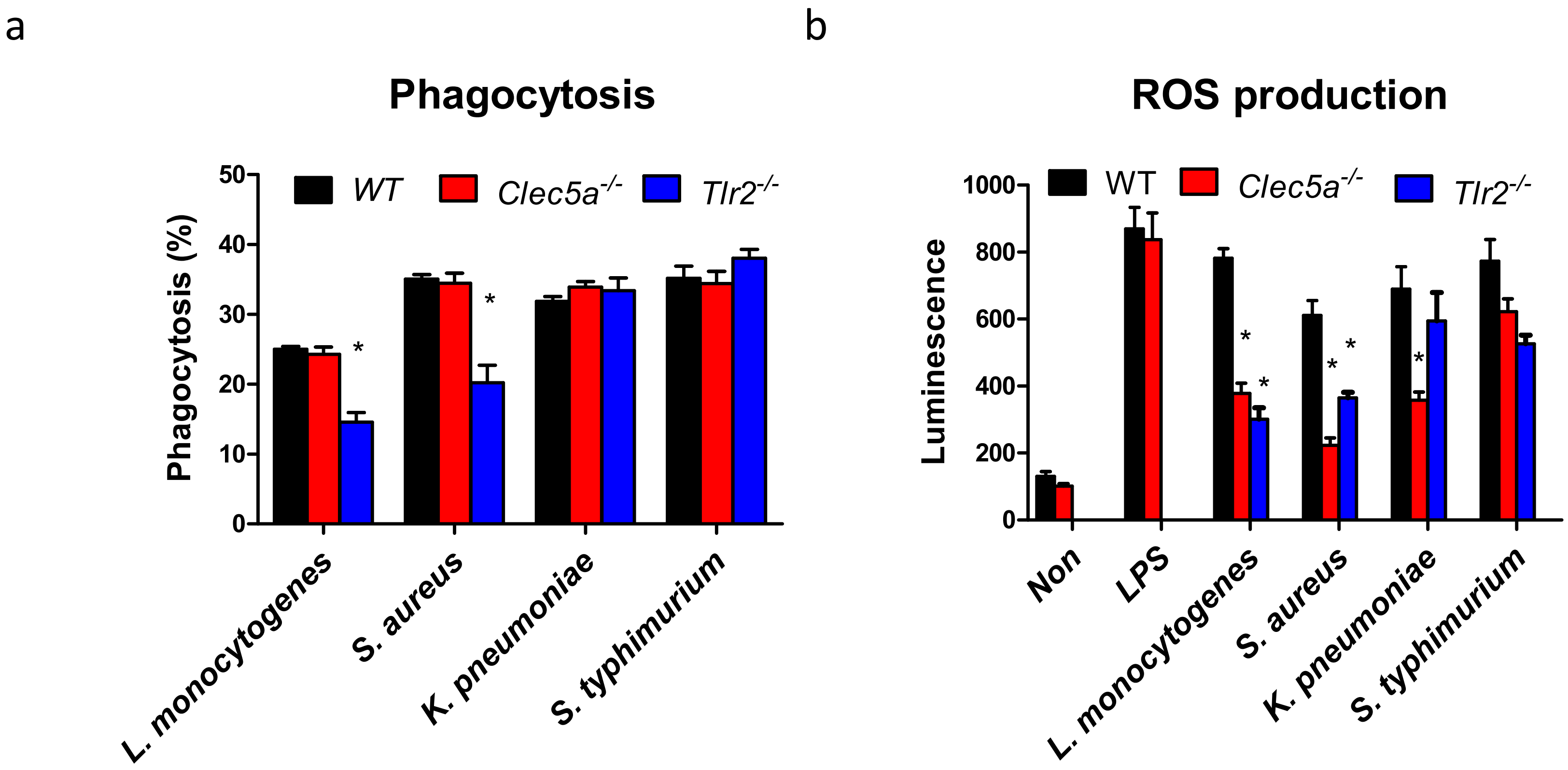

Supplementary Figure 2. CLEC5A participates in ROS production, but not in phagocytosis, after incubation with *L. monocytogenes.* (**a**) Mouse neutrophils were incubated with live bacteria (MOI 0.1) for 60 min to determine phagocytic ability. (**b**) ROS production was measured by luminescence at 10-min intervals for 120 min. The representative ROS production in each group is shown as the luminescence intensity at 60 min post *L. monocytogenes* infection (MOI 3). Data were collected and expressed as mean $\pm$ s.e.m. from at least three independent experiments. One-way ANOVA was performed. *P<0.05 for wild type versus knockout mice.

Supplementary Figure 3

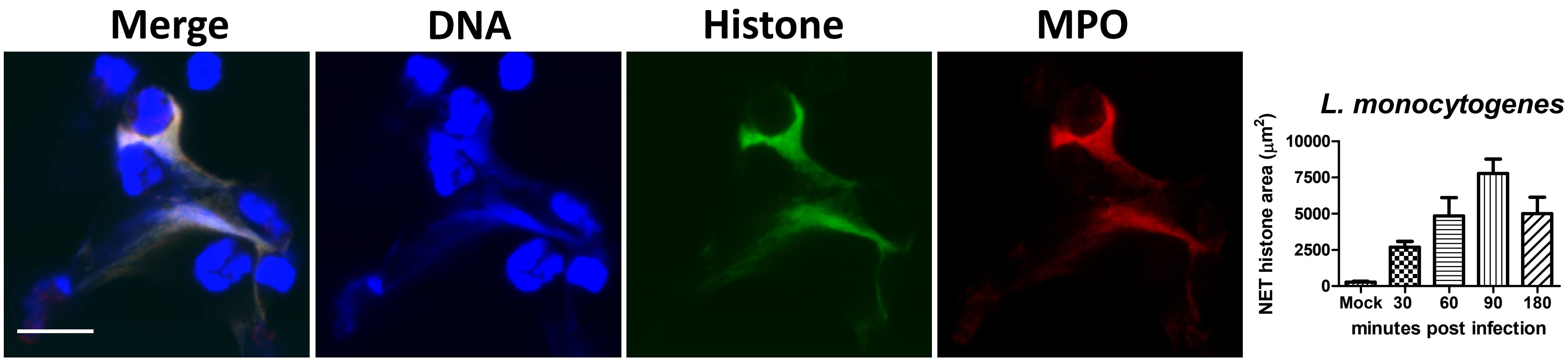

Supplementary Figure 3. Mouse NET formation induced by *L. monocytogenes*. Mouse neutrophils were incubated with live *L. monocytogenes* (MOI 10),and NET structures were observed at indicated time points post infection by immunofluorescence staining using anti-MPO (red), anti-histone (green) Abs and Hoechst 33342 (blue) with a confocal microscope. Scale bar, 10 μm. The NET quantification was displayed as NET histone area (μm^2) per filed. Five independent fields were photographed to measure the histone area. Data were collected and expressed as mean ± s.e.m. from at least three independent experiments.

Supplementary Figure 4

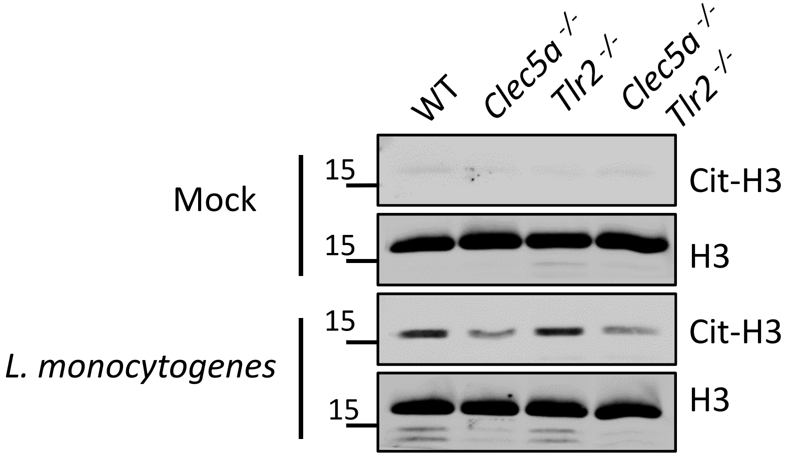

Supplementary Figure 4. Deficiency of CLEC5A results in inefficient induction of histone 3 citrullination in neutrophils.
Mouse neutrophils were incubated with live *L. monocytogenes* (MOI 10) and lysates were harvested at 60 min to determine the levels of citrullinated histone 3 (Cit-H3) and internal loading control histone 3 (H3).

Supplementary Figure 5

a

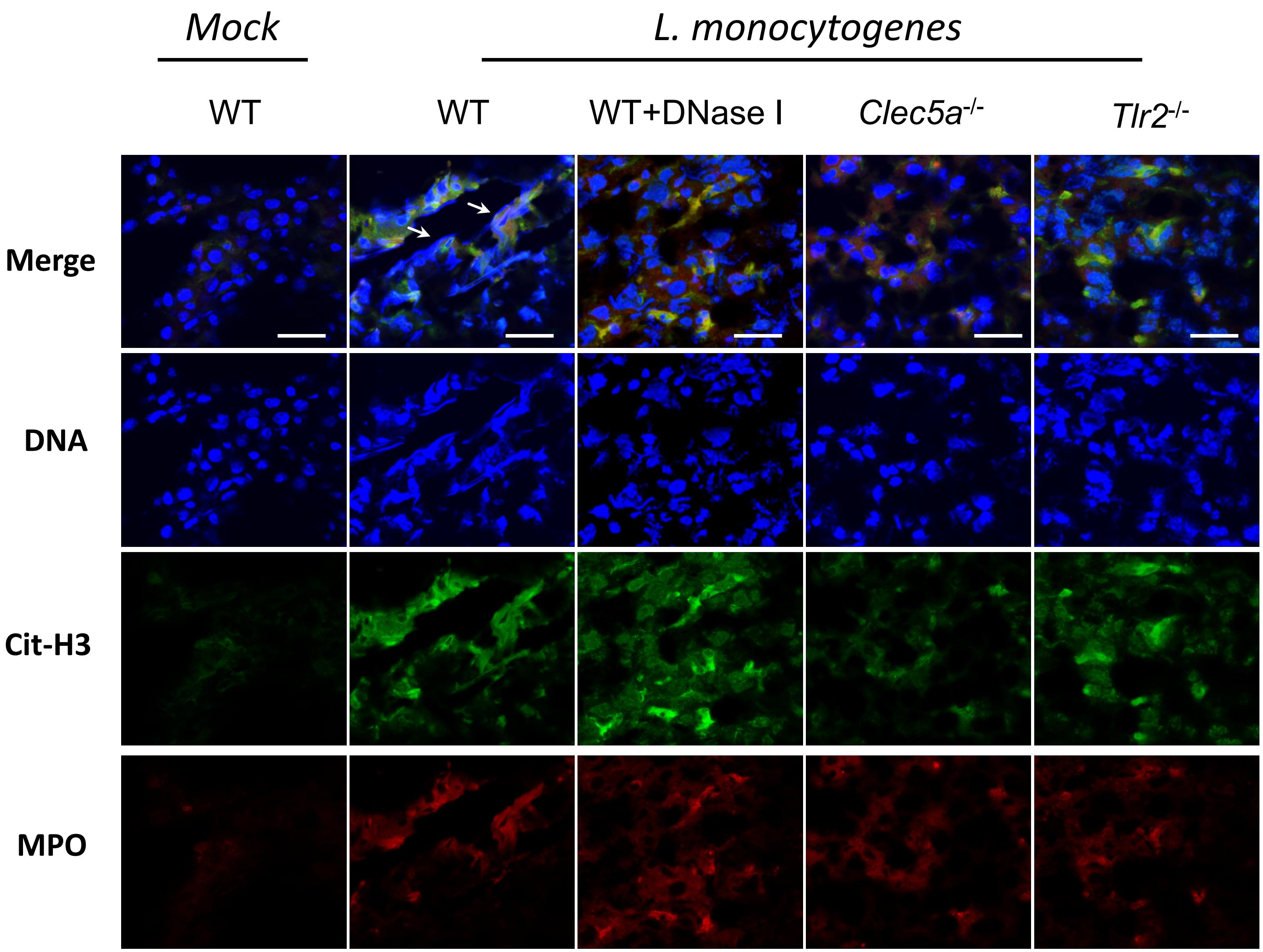

Supplementary Figure 5

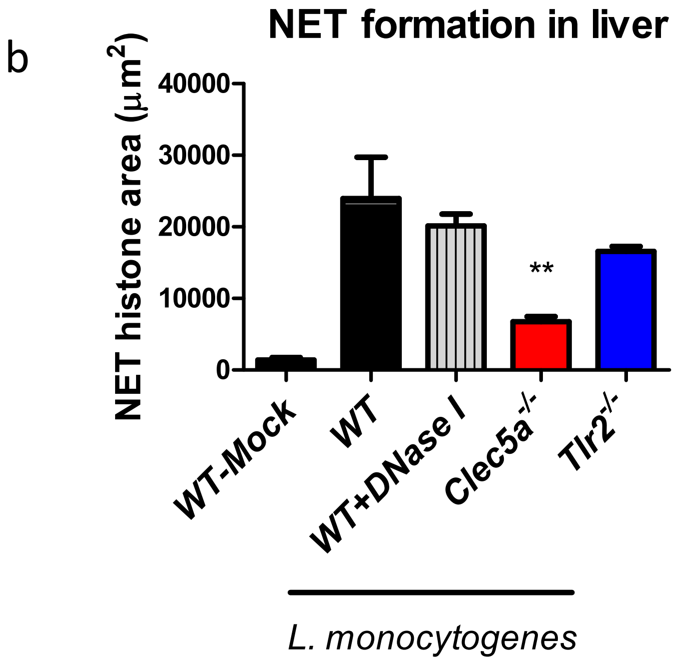

Supplementary Figure 5. CLEC5A and TLR2 are involved in NET formation in vivo.
(**a**) Representative images of NET release (arrows) in the liver of WT, WT/DNase-I, and mutant mice at 4 h post intravenous inoculation of *L. monocytogenes* (3×10^8 CFUs/mouse, Xen32; n=3 per group). NET release was determined by immunofluorescence staining with antibodies against citrullinated histone 3 (Cit-H3, green) and MPO (red), and counterstaining with Hoechst 33342 for DNA (blue), followed by observation with a confocal microscopy. Scale bar, 20 μm. (**b**) The NET quantification was displayed as the NET histone area (μm^2) /per filed. Data were collected and expressed as mean ± s.e.m. from at least three independent experiments. One-way ANOVA was performed. ***P*<0.01; for WT versus knockout mice.

Supplementary Figure 6

a

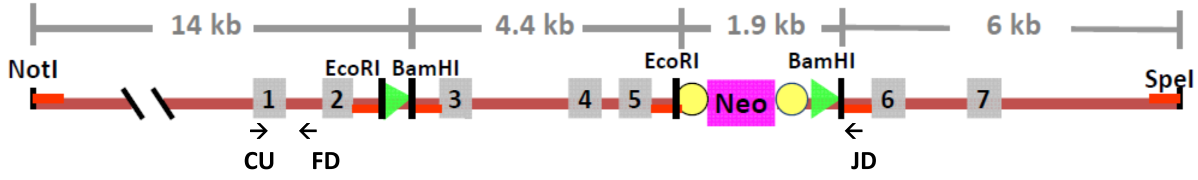

b

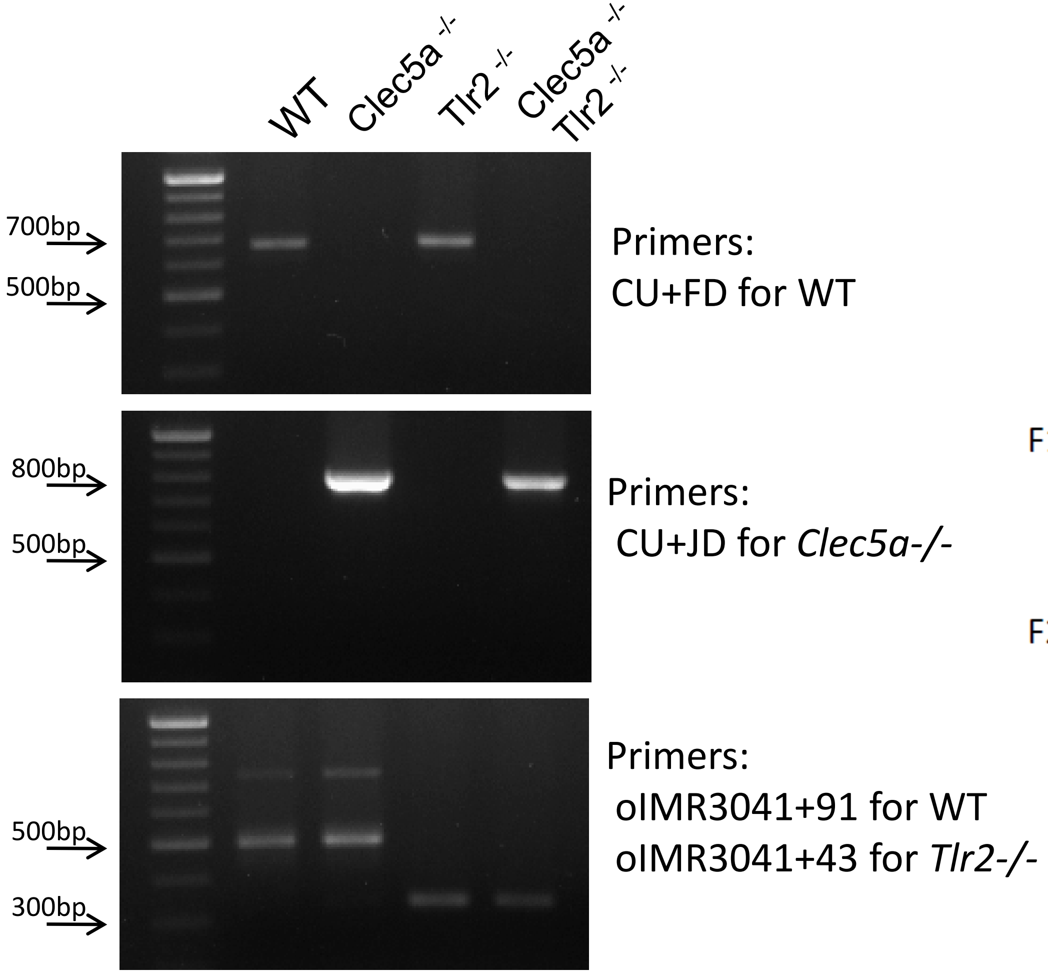

c

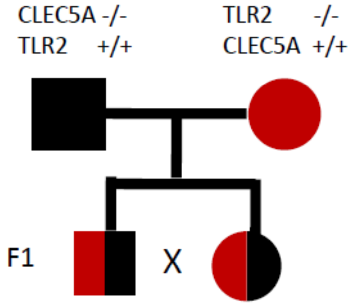

F1

CLEC5A	+	-
+	++	-+
-	+-	- -

TLR2	+	-
+	++	-+
-	+-	- -

F2

CLEC5A	TLR2	CLEC5A	TLR2	CLEC5A	TLR2	CLEC5A	TLR2
++	++	--	++	+-	++	+-	++
++	+-	--	+-	+-	+-	+-	+-
++	+-	--	+-	+-	+-	+-	+-
++	- -	--	- -	+-	- -	+-	- -

Supplementary Figure 6. Strategy for generation of CLEC5A and TLR2 double knockout mice. (**a**) Targeting vector of generation of *Clec5a* $^{-/-}$ mice. The locations of PCR primers used for genotyping are shown under the targeting vector. (**b**) Genotyping of knockout mice was performed by PCR using CU+FD and CU+JD primer sets to identify the wild type (WT) and *Clec5a*$^{-/-}$ mice, respectively. The sequences of primers used to distinguish WT and *Tlr2*$^{-/-}$ mice were from the official website of Jackson Laboratory. (**c**) Double knockout mice were produced by mating *Clec5a*$^{+/+}$/*Tlr2*$^{-/-}$ and *Clec5a*$^{-/-}$/*Tlr2*$^{+/+}$ mice, and the F1 offspring were interbred to generate F2 offspring.

Supplementary Figure 7

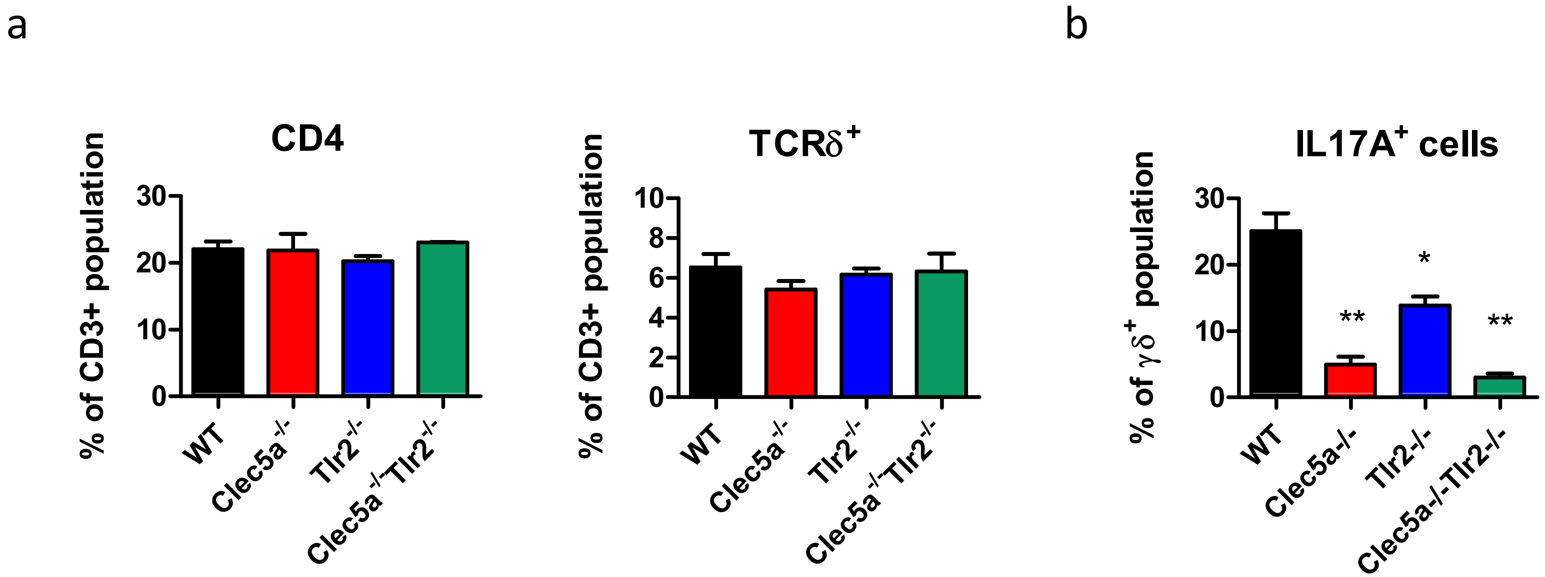

Supplementary Figure 7. Deficiency of CLEC5A results in a reduction of IL-17A-producing TCRγδ$^+$ T cells in response to *L. monocytogenes* infection. All groups of mice were intravenously challenged with 1× 10^5 CFUs of *L. monocytogenes (*10403S), and liver mononuclear cells were isolated to analyze the T cell populations at day 5 post infection by the FACS method. (**a**) The percentage of CD4$^+$ and TCRγδ$^+$ T cells over the total CD3$^+$ T cells were calculated. (**b**) The percentage of IL-17A producing cells over total TCRγδ$^+$ T cell population were calculated. Data were collected and expressed as mean ± s.e.m. from at least three independent experiments. One-way ANOVA was performed. *P<0.05, *P<0.01 for WT versus knockout mice.

Supplementary Figure 8

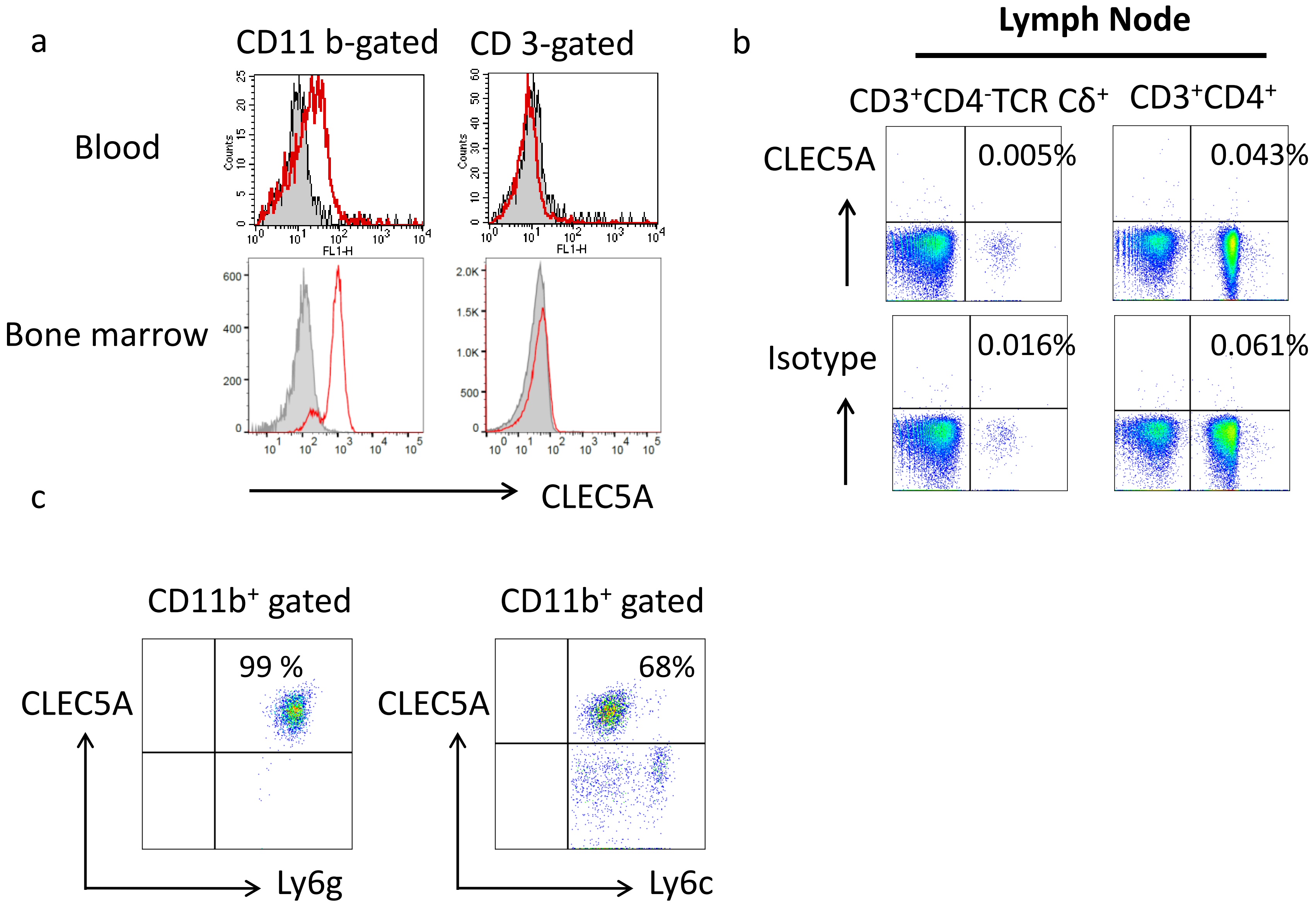

Supplementary Figure 8. Expression of CLEC5A in mouse immune cells.

(**a**) Freshly isolated mouse blood and bone marrow cells were double stained with fluorochrome–conjugated antibodies to CD markers and APC-conjugated anti-mouse CLEC5A mAb. CD marker positive cells were gated to determine the expression of CLEC5A (red lines). Shaded areas represent isotype controls. (**b**) Cells isolated from mouse lymph nodes were determined the CLEC5A expression in the subpopulation of T cells including γδ T ($CD3^+CD4^-$ TCR $γδ^+$) and CD4 Tcells ($CD3^+CD4^+$TCR $γδ^-$). (**c**) Cells isolated from mouse bone marrow were determined the CLEC5A expression in the subpopulation of cells of myeloid lineage including neutrophils ($CD11b^+Ly6g^+$) and monocytes ($CD11b^+Ly6c^+$).

Supplementary Figure 9

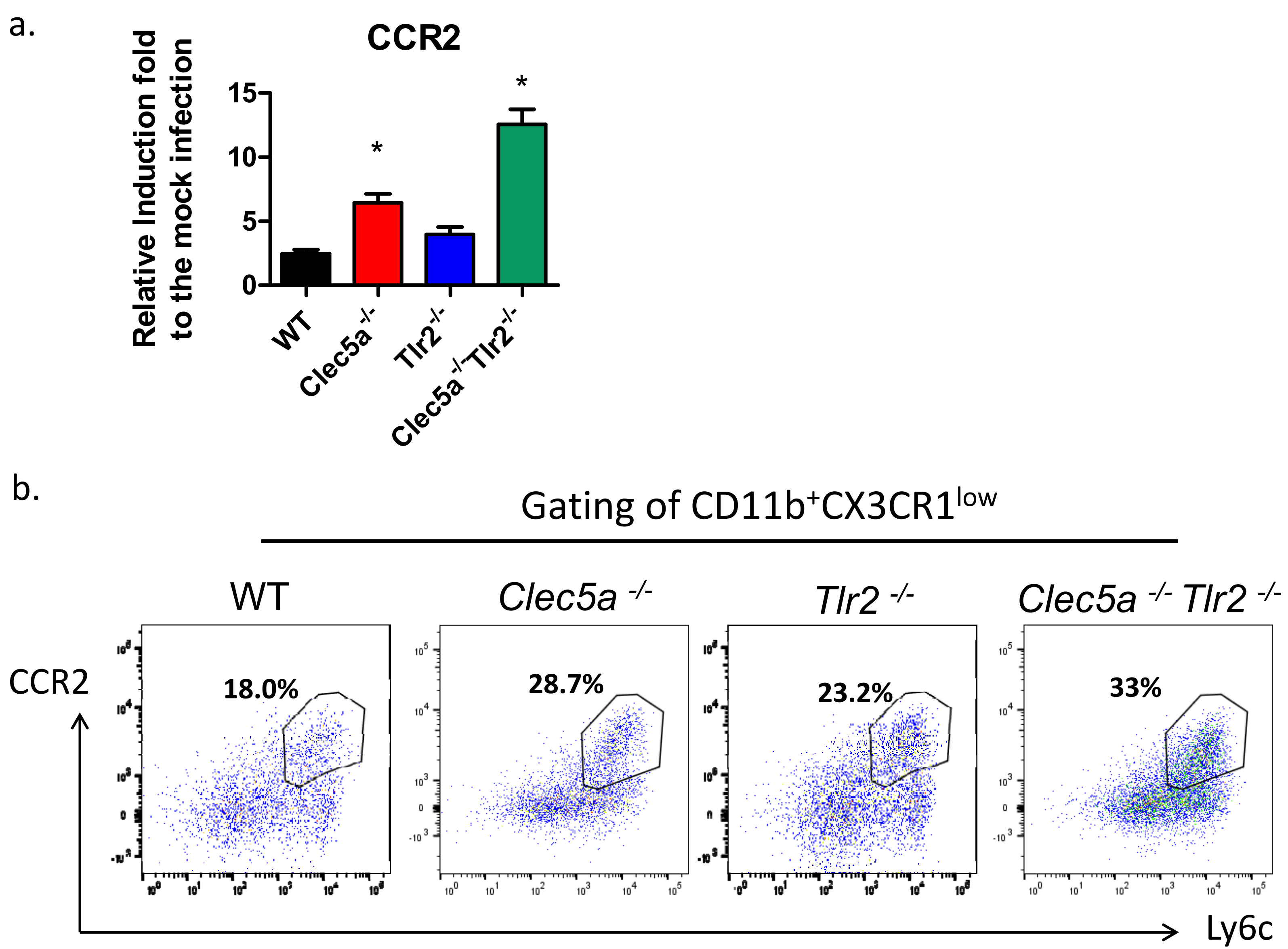

Supplementary Figure 9. Deficiency of CLEC5A results in increased recruitment of CCR2$^+$ inflammatory monocytes in liver after *L. monocytogenes* infection. All groups of mice were intravenously challenged with 1$\times$ 10^5 CFUs of *L. monocytogenes (*10403S). (**a**) Livers were harvested at day 5 post infection for determining the *ccr2* expression by q-PCR. Data were collected and expressed as mean $\pm$ s.e.m. from at least three independent experiments. One-way ANOVA was performed. **P*<0.05 for WT versus knockout mice (**b**) Population of inflammatory monocytes (CD11b$^+$CCR2$^+$Ly6c^{hi}CX3CR1^{low}) was determined using the markers of CD11b, CX3CR1, Ly6c and CCR2. The representative FACS data shows the percentage of liver CCR2$^+$Ly6c^{hi} cells under a CD11B$^+$CX3CR1^{low} gating condition.

Supplementary Figure 10

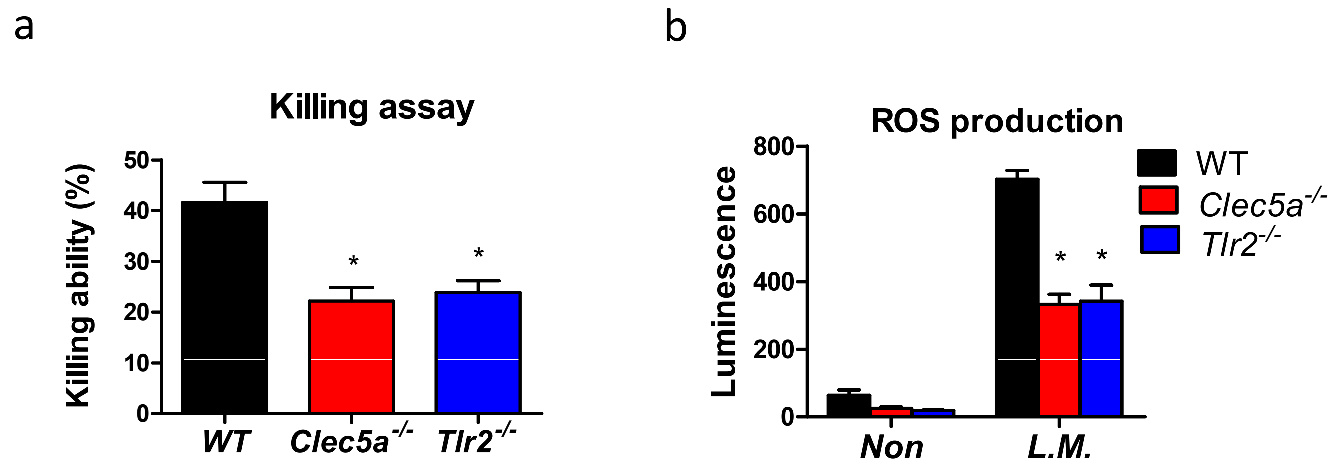

Supplementary Figure 10. Deficiency of CLEC5A leads to functional impairment in bone marrow-derived macrophages. Mouse macrophages were incubated with live *L. monocytogenes* (MOI 0.1) for 60 min to determine (**a**) killing ability. (**b**) For ROS measurement, luminescence was measured at 10-min intervals for 120 min. The representative ROS production in each group is shown as the luminescence intensity at 60 min post *L. monocytogenes* infection (MOI 3) Data were collected and expressed as mean ± s.e.m. from at least three independent experiments. One-way ANOVA was performed. *P<0.05 for WT versus knockout mice.

Supplementary Figure 11

a

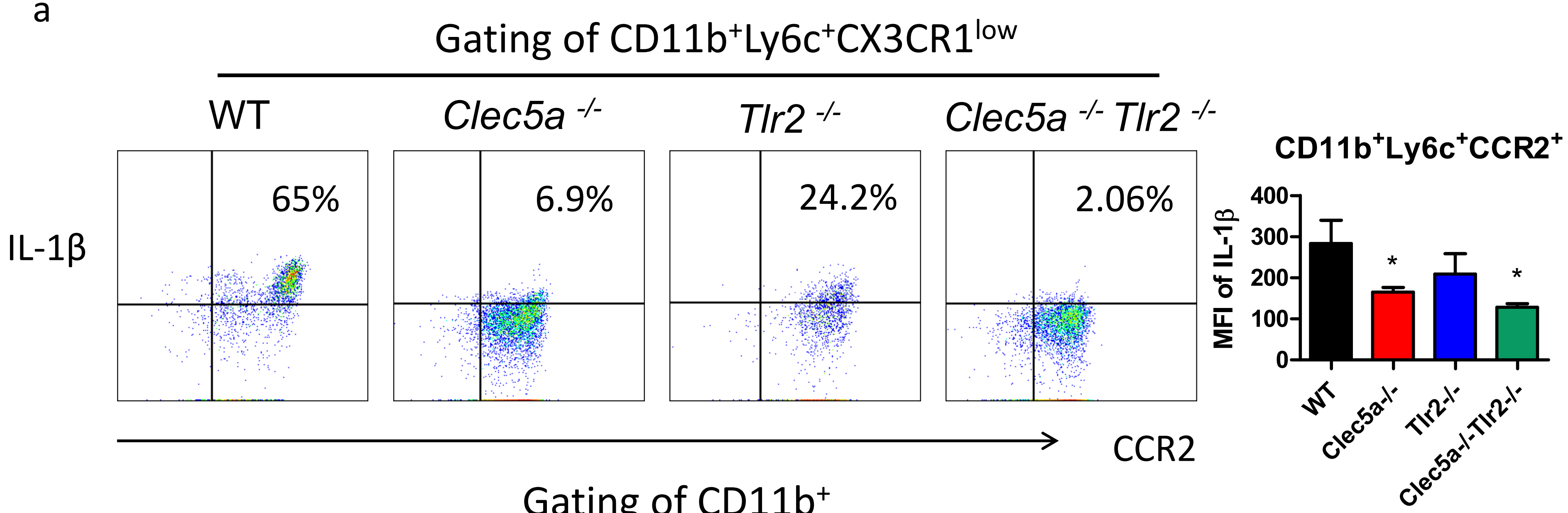

b

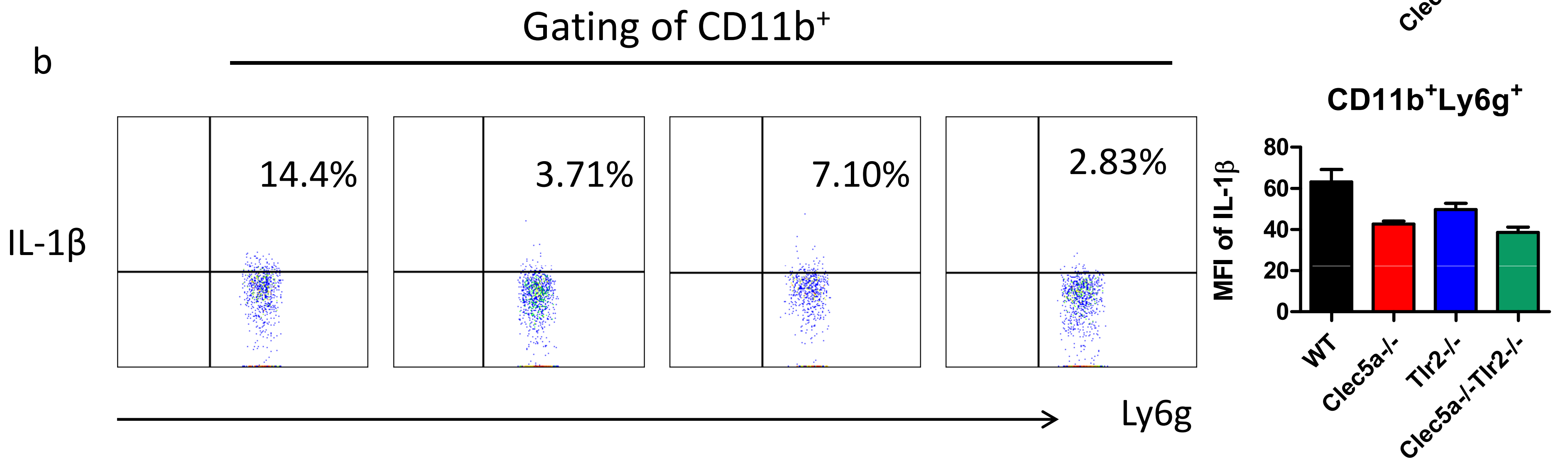

Supplementary Figure 11. Deficiency of CLEC5A results in impairment of IL-1β expression in the $CCR2^+$ inflammatory monocytes and $Ly6g^+$ neutrophils in response to *L. monocytogenes* infection. All groups of mice were intravenously challenged with 1×10^5 CFUs of *L. monocytogenes (*10403S). IL-1β expression in the population of (**a**) inflammatory monocytes ($CD11b^+CCR2^+Ly6c^{hi}CX3CR1^{low}$) and (**b**) neutrophils ($CD11b^+Ly6g^+$) was determined using the markers CCR2, CD11b, CX3CR1, Ly6c and Ly6g, followed by intracellular staining with IL-1β antibody. The representative FACS data are shown and the mean florescence intensity (MFI) of IL-1β is shown in the histograms. Data were collected and expressed as mean $\pm$ s.e.m. from at least three independent experiments. One-way ANOVA was performed. *$P<0.05$ for WT versus knockout mice.

Supplementary Figure 12

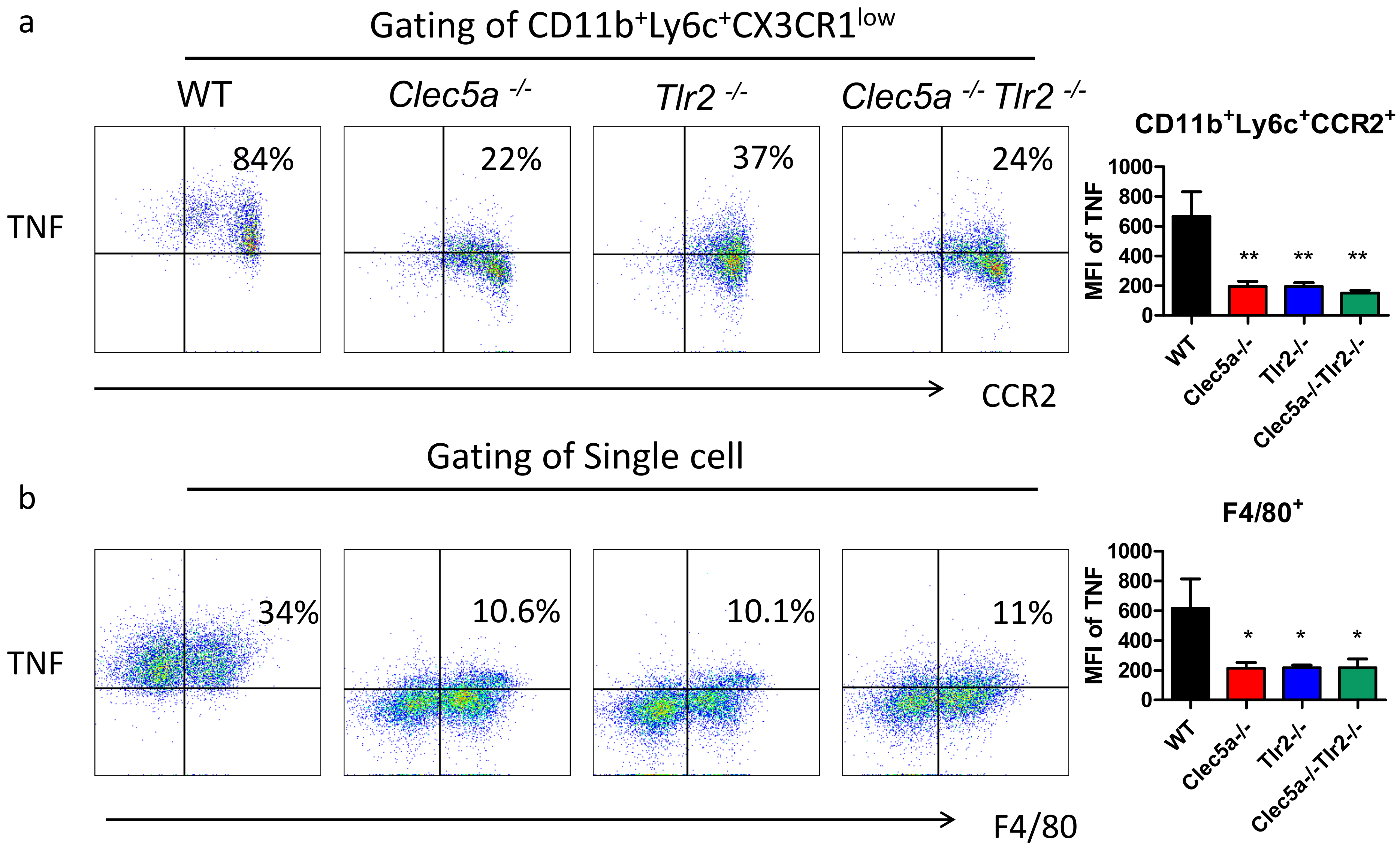

Supplementary Figure 12. Deficiency of CLEC5A results in impairment of TNF expression in the CCR2^{+} inflammatory monocytes and F4/80^{+} macrophages in response to *L. monocytogenes* infection. All groups of mice were intravenously challenged with 1×10^{5} CFUs of *L. monocytogenes (*10403S). TNF-α expression in the population of (**a**) inflammatory monocytes (CD11b^{+}CCR2^{+}Ly6c^{hi}CX3CR1^{low}) and (**b**) macrophages (CD11b^{+}F4/80^{+}) was determined using the markers CCR2, CD11b, CX3CR1, Ly6c and F4/80, followed by intracellular staining with TNF antibody. The representative FACS data were shown and the mean florescence intensity (MFI) of TNF is shown in the histograms. Data were collected and expressed as mean $\pm$ s.e.m. from at least three independent experiments. One-way ANOVA was performed. *$P<0.05$; **$P<0.01$ for WT versus knockout mice.

Supplementary Figure 13

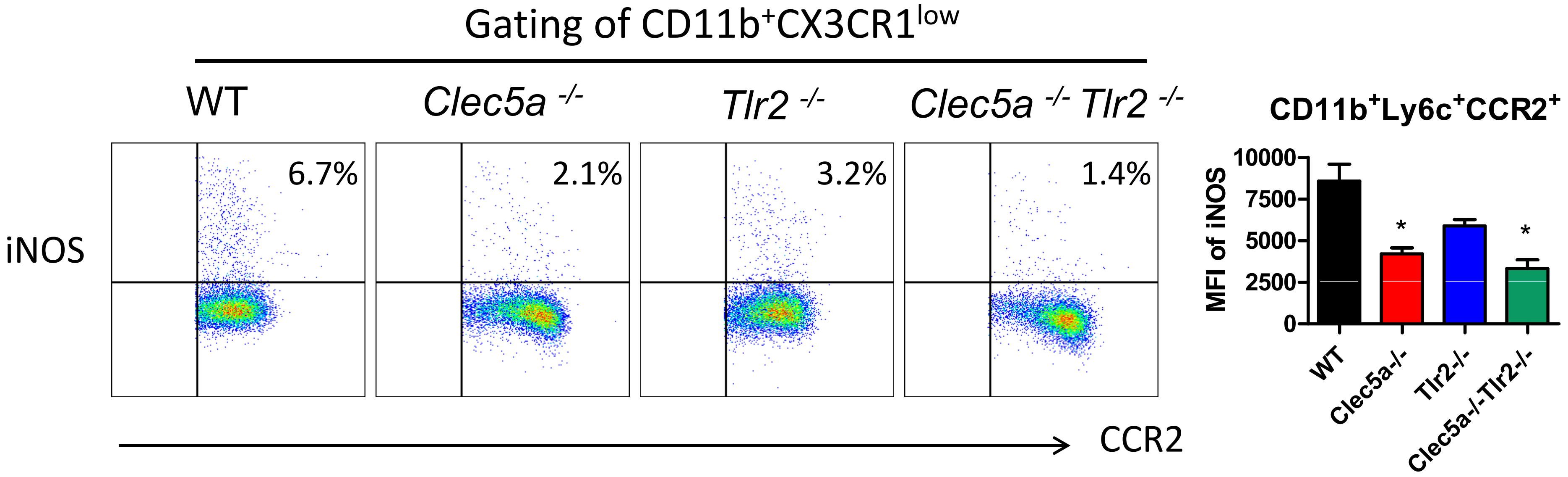

Supplementary Figure 13. Deficiency of CLEC5A results in impairment of iNOS expression in the CCR2$^+$ inflammatory monocytes in response to *L. monocytogenes* infection.

All groups of mice were intravenously challenged with 1× 10^5 CFUs of *L. monocytogenes (*10403S). iNOS expression in the population of inflammatory monocytes (CD11b$^+$CCR2$^+$Ly6c^{hi}CX3CR1^{low}) was determined using the markers CCR2, CD11b, CX3CR1 and Ly6c, followed by intracellular staining with iNOS antibody. The representative FACS data are shown and the mean florescence intensity (MFI) of iNOS is shown in the histograms.. Data were collected and expressed as mean ± s.e.m. from at least three independent experiments. One-way ANOVA was performed. *P<0.05 for WT versus knockout mice.

Supplementary Figure 14

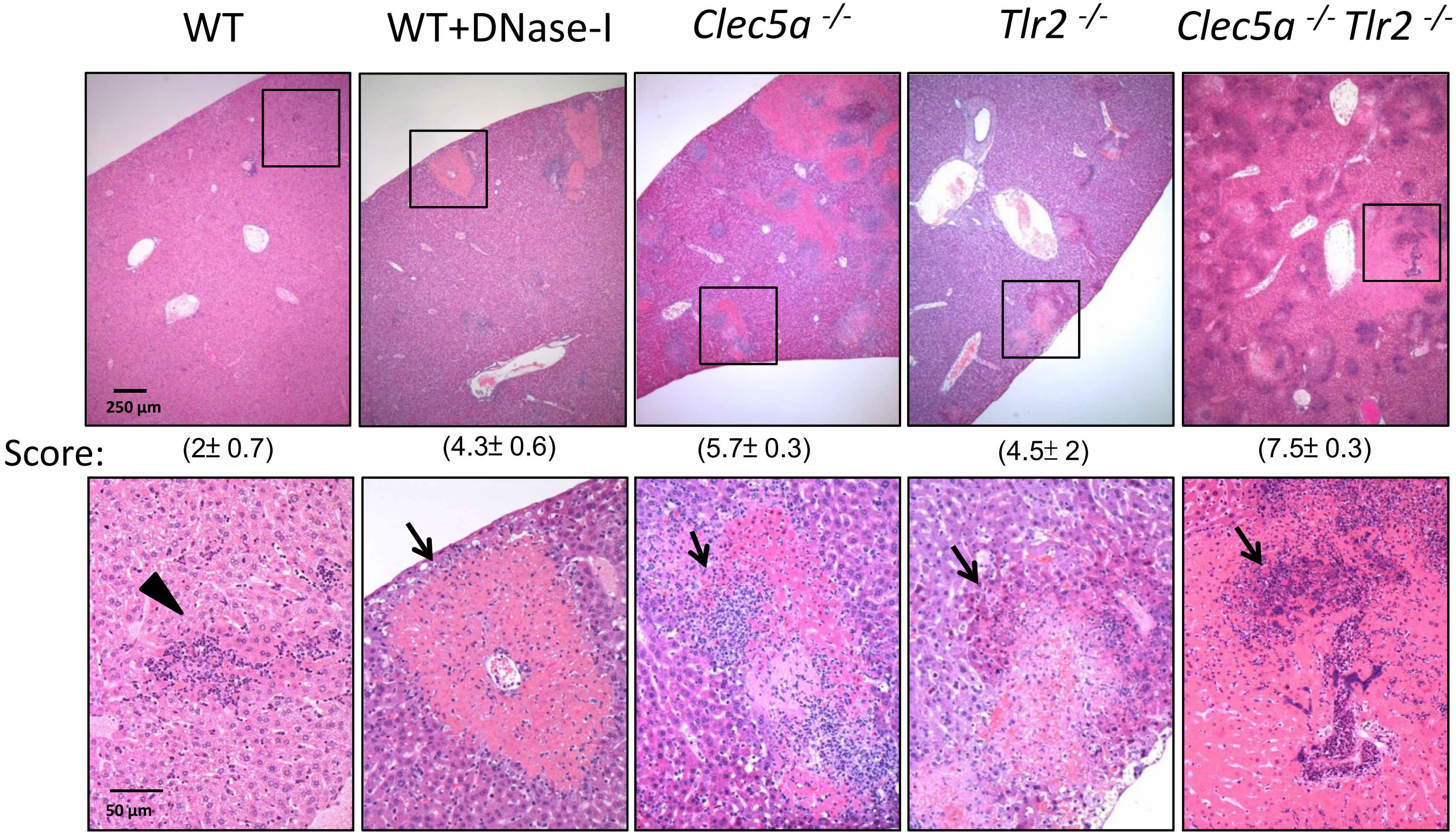

Supplementary Figure 14. Deficiency of CLEC5A and TLR2 results in severe liver damage after *L. monocytogenes* infection. All groups of mice were intravenously challenged with 1×10^5 CFUs of *L. monocytogenes (*10403S). Livers were harvested at day 5 post infection and processed for H&E staining. *Upper panel*: representative low power field of view images. The degree of liver inflammation (mean $\pm$ SEM) was calculated as described in the Methods section and is annotated under the panel. *Lower panel*: high power field of view images from the highlighted regions in the upper panel. Arrowhead: abscess. Arrow: region of necrosis (5 mice per group).

Supplementary Figure 15

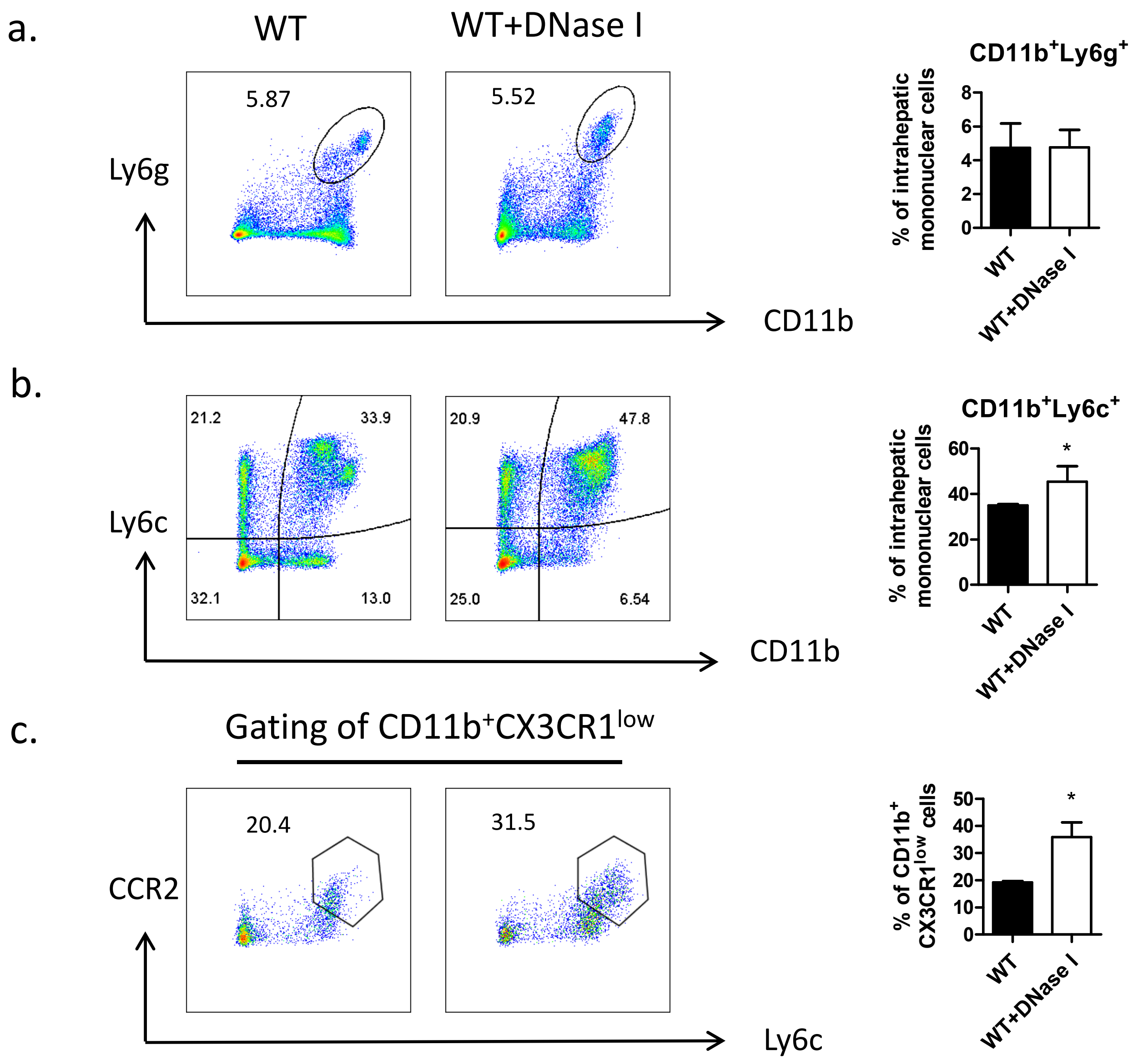

Supplementary Figure 15. Removal of NETs by DNase I results in increased recruitment of $CD11b^+Ly6c^+$ monocytes and $CCR2^+$ inflammatory monocytes post *L. monocytogenes* infection. All groups of mice were intravenously challenged with 1×10^5 CFUs of *L. monocytogenes (*10403S). To determine the effect of NET formation during *L. monocytogenes* infection, mice were injected intraperitoneally with DNase I (2 KU) every 2 days. Livers were harvested to determine the population of (**a**) $CD11b^+Ly6g^+$ neutrophils, (**b**) $CD11b^+Ly6c^+$ monocytes and (**c**) $CD11b^+CCR2^+Ly6c^{hi}CX3CR1^{low}$ inflammatory monocytes at day 5 post- *L. monocytogenes* infection. Right: Data were collected from three independent experiments (n=5 for each group). Student's *t* test was performed. *P<0.05 for WT versus DNase I-treated mice.

Supplementary Figure 16

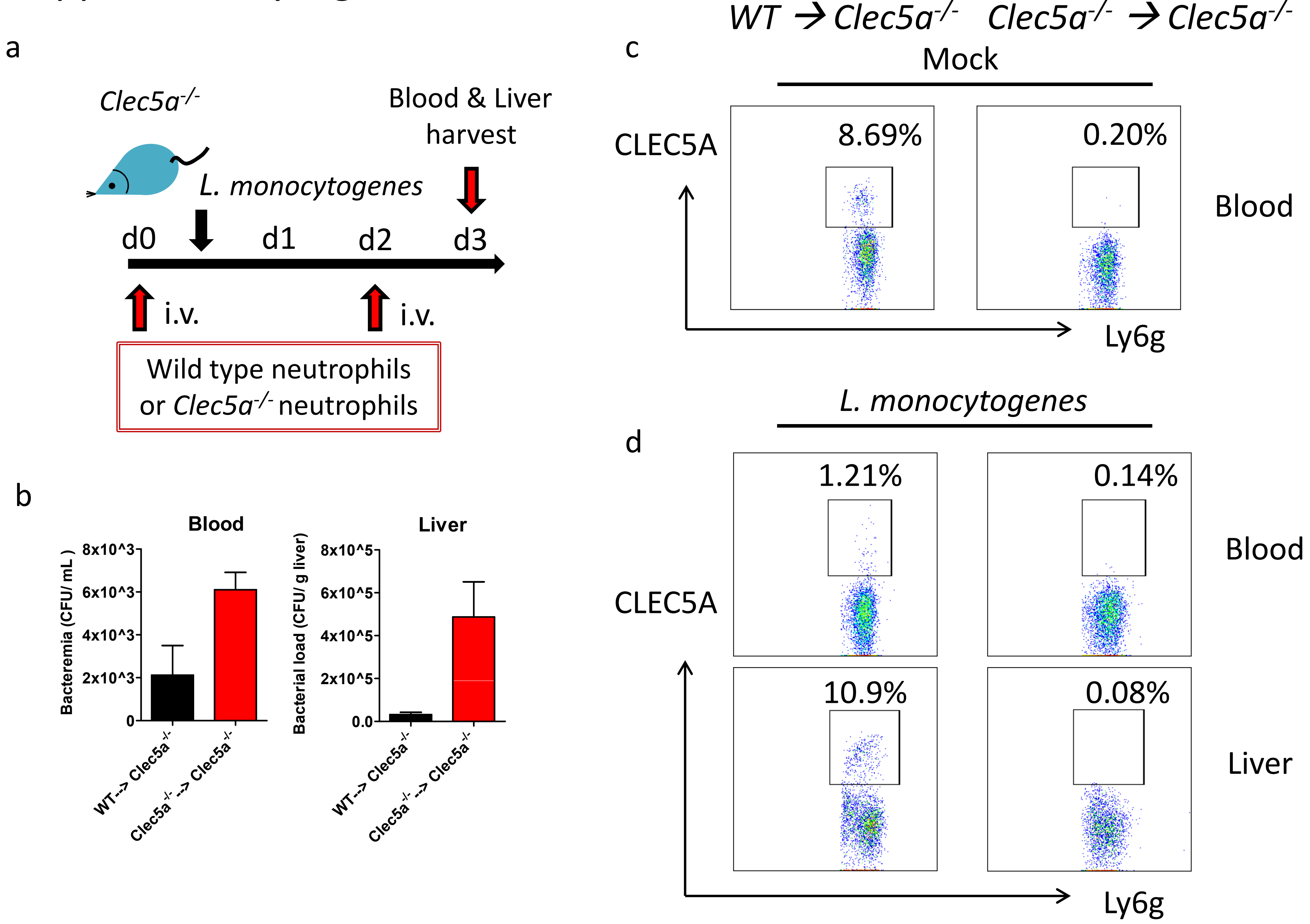

Supplementary Figure 16. Inoculation of wild type neutrophils protects CLEC5A knockout mice from Listeria infection. (**a**) Flow chart is shown the adoptive transfer of wide type or *Clec5a*$^{-/-}$ neutrophils into *Clec5a*$^{-/-}$ mice. At day 0, neutrophils (6×10^6) from the WT and *Clec5a*$^{-/-}$ mice were intravenously injected into *Clec5a*$^{-/-}$ mice, followed by intravenously inoculating *L. monocytogenes* (1×10^5 CFUs). At day 2, neutrophils (6×10^6) were injected in *Clec5a*$^{-/-}$ mice and these mice were sacrificed at day 3 post infection. (**b**) Blood and livers were harvested to determine bacterial loads. (**c**) Neutrophils reconstitution rate of WT neutrophils in the blood of *Clec5a*$^{-/-}$ mice. Blood cells from the recipient mice were incubated with fluorochrome-conjugated anti-Ly6g, anti-CD11b, and anti-CLEC5A mAbs, and the percentage of CLEC5A$^+$ cells in the CD11b$^+$ and Ly6g$^+$ population was determined by the flow cytometry. (**d**) Cells were harvested from the livers of *Clec5a*$^{-/-}$ mice at day 3 post-infection, and CLEC5A$^+$CD11b$^+$ Ly6g$^+$ neutrophil populations were determined by the flow cytometry.

Supplementary Figure 17

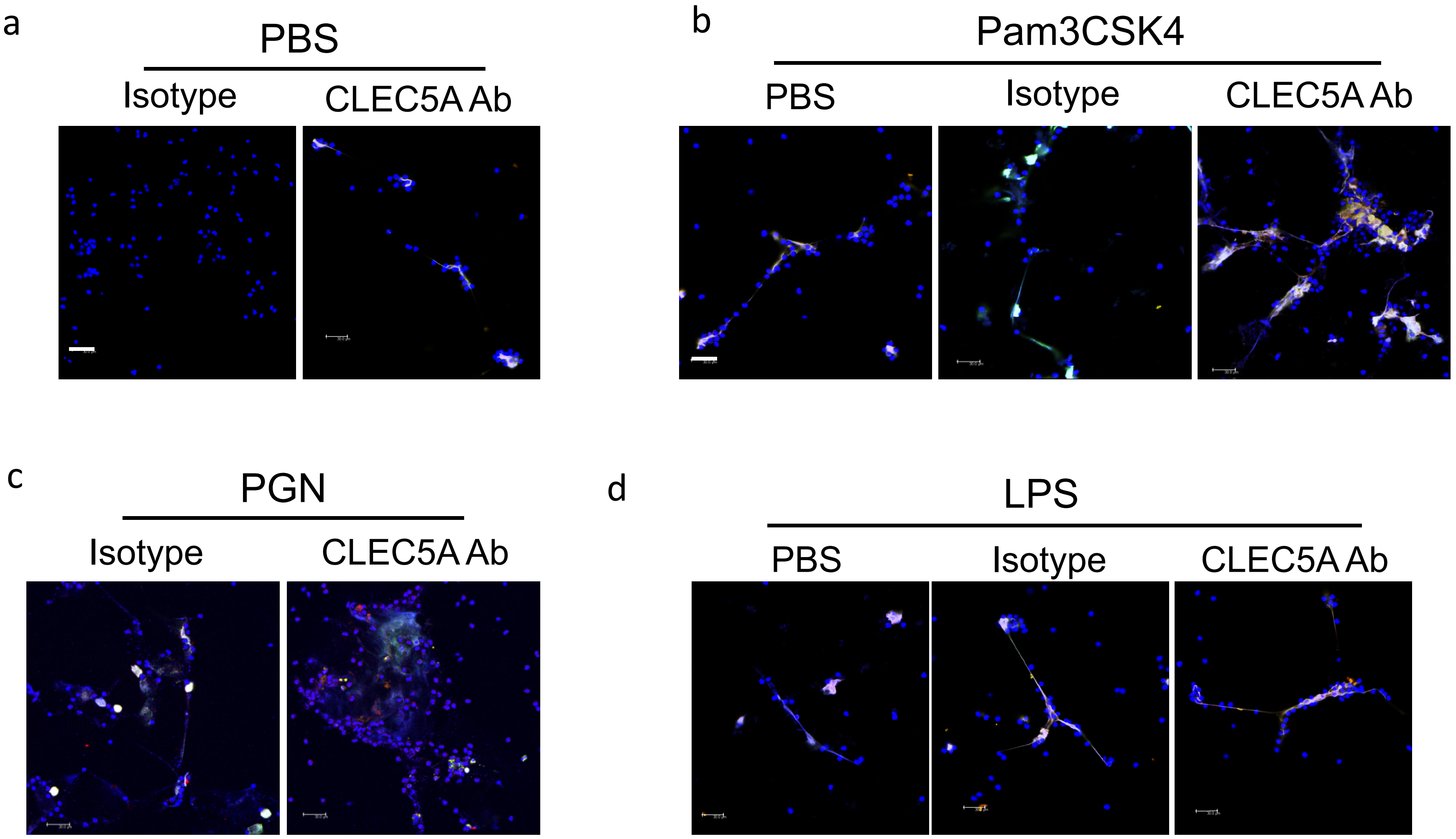

Supplementary Figure 17. Coactivation of CLEC5A and TLR2 promotes NET formation. Human neutrophils were incubated with (**a**) agonistic anti-CLEC5A mAb (2D8G9) alone, or in conjunction with (**b**) Pam3CSK4, (**c**) peptidoglycans (PGN), or (**d**) lipopolysaccharide (LPS) for 2 h. NET components were detected by immunofluorescence staining using antibodies against MPO (red), citrullinated histone H3 (green) and Hoechst 33342 (blue), followed by observation with a confocal microscope. Scale bar, 30 μm.

Supplementary Figure 18

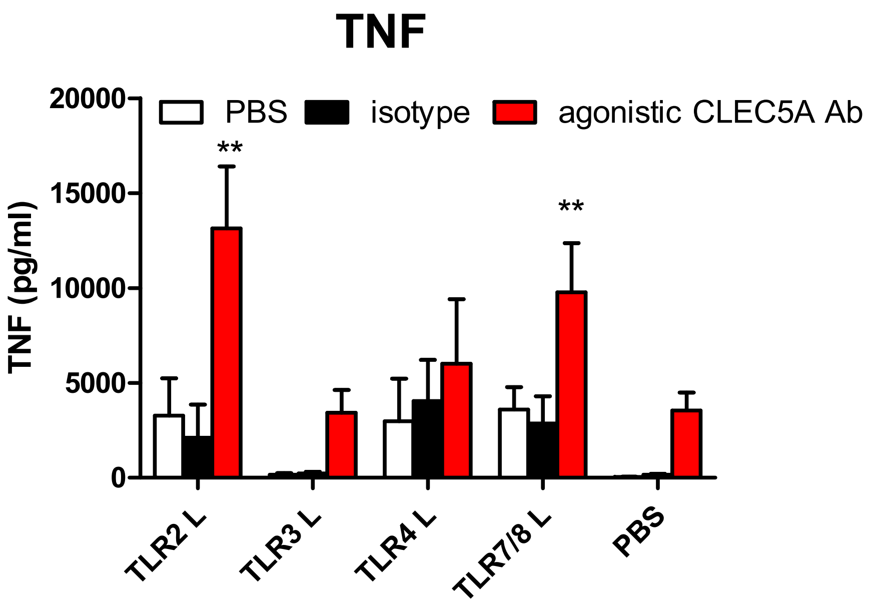

Supplementary Figure 18. Coactivation of CLEC5A and TLR promotes TNF production. Human primary macrophages were incubated with agonistic anti-CLEC5A mAb (2D8G9) for 30 min, followed by crosslinking with goat anti-mouse IgG antibody for 2 h. TLR ligands including TLR2 L (Pam3CSK4, 1 μg/ml), TLR3 L (Poly I:C, 10 μg/ml), TLR4 L (LPS, 1 μg/ml) and TLR7/8 L (CL097, 1 μg/ml) were added to the CLEC5A antibody-treated macrophages for another 24 h. TNF secretion was measured by ELISA. Data were collected and expressed as mean ± s.e.m. from three independent experiments. One-way ANOVA was performed. ***P*<0.01 for anti-CLEC5A mAb treatment versus isotype control.

Supplementary Figure 19

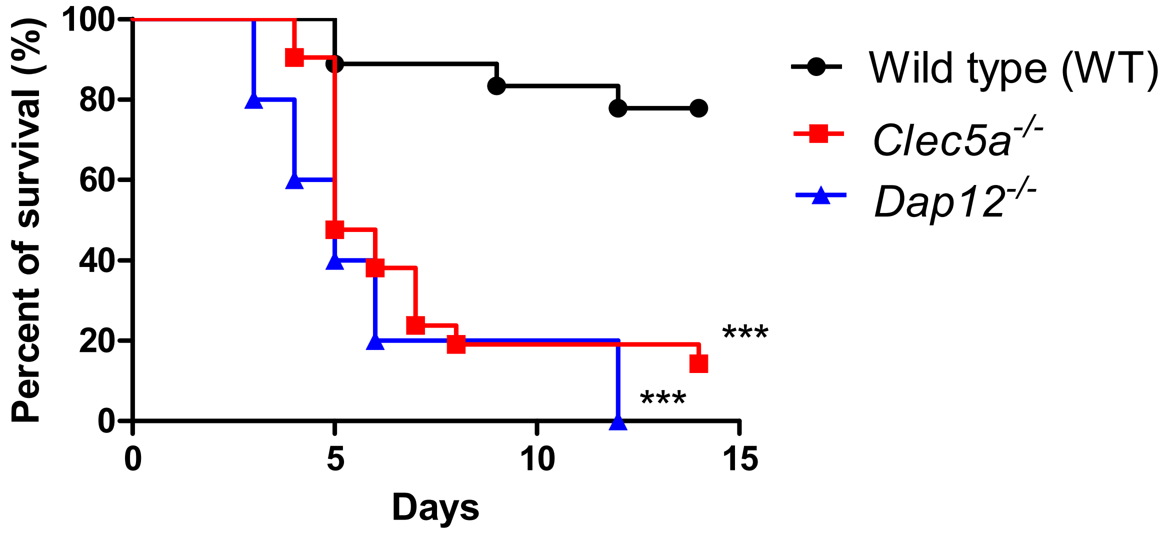

Supplementary Figure 19. CLEC5A and DAP12 deficiency results in high mortality rate post *L. monocytogenes* infection. Mice were intravenously challenged with 1× 10^5 CFUs of *L. monocytogenes (*10403S), and survival rate was monitored daily for 15 days. Data were collected from four independent experiments, and the percentage of survived mice is shown as Kaplan–Meier survival curves with log rank test (n = 8 for each group).

Supplementary Figure 20

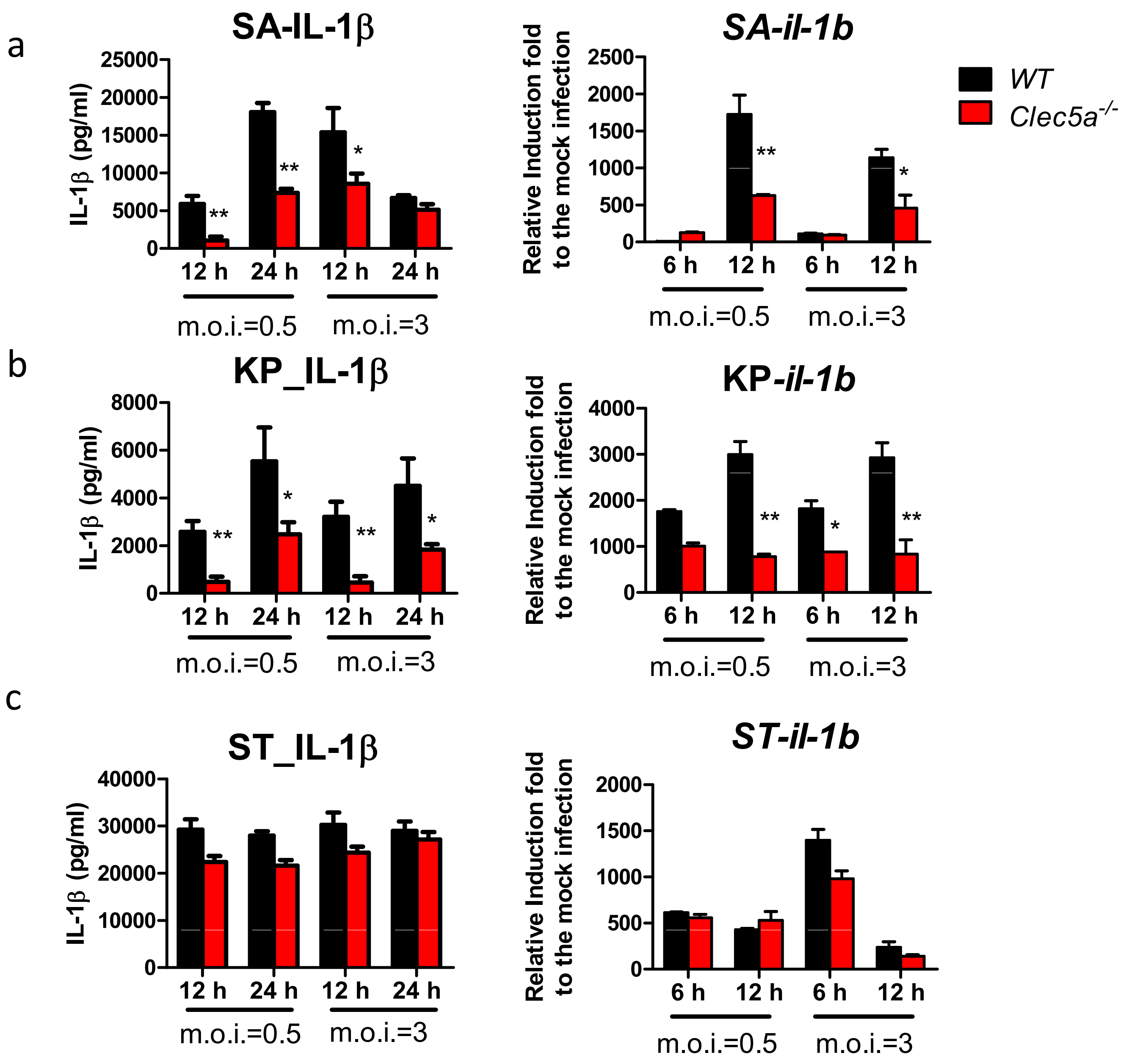

Supplementary Figure 20. CLEC5A is involved in *Staphylococcus aureus* and *Klebsiella pneumoniae*-induced IL-1β production. Mouse macrophages were incubated with live *Staphylococcus aureus* (SA)*, Klebsiella pneumoniae* (KP) *and Salmonella typhimurium* (ST), respectively, and supernatants were harvested, and IL-1β secretion was measured by ELISA. The levels of *il-1b* transcription were detected by RT-qPCR. All data were collected and expressed as mean ± s.e.m. from at least three independent experiments. Student's *t* test was performed. **P*<0.05, ***P*<0.01 for wild type versus CLEC5A knockout cells. (**a**) *Staphylococcus aureus*; (**b**) *Klebsiella pneumoniae* and (**c**) *Salmonella typhimurium*.

Supplementary Fig 21

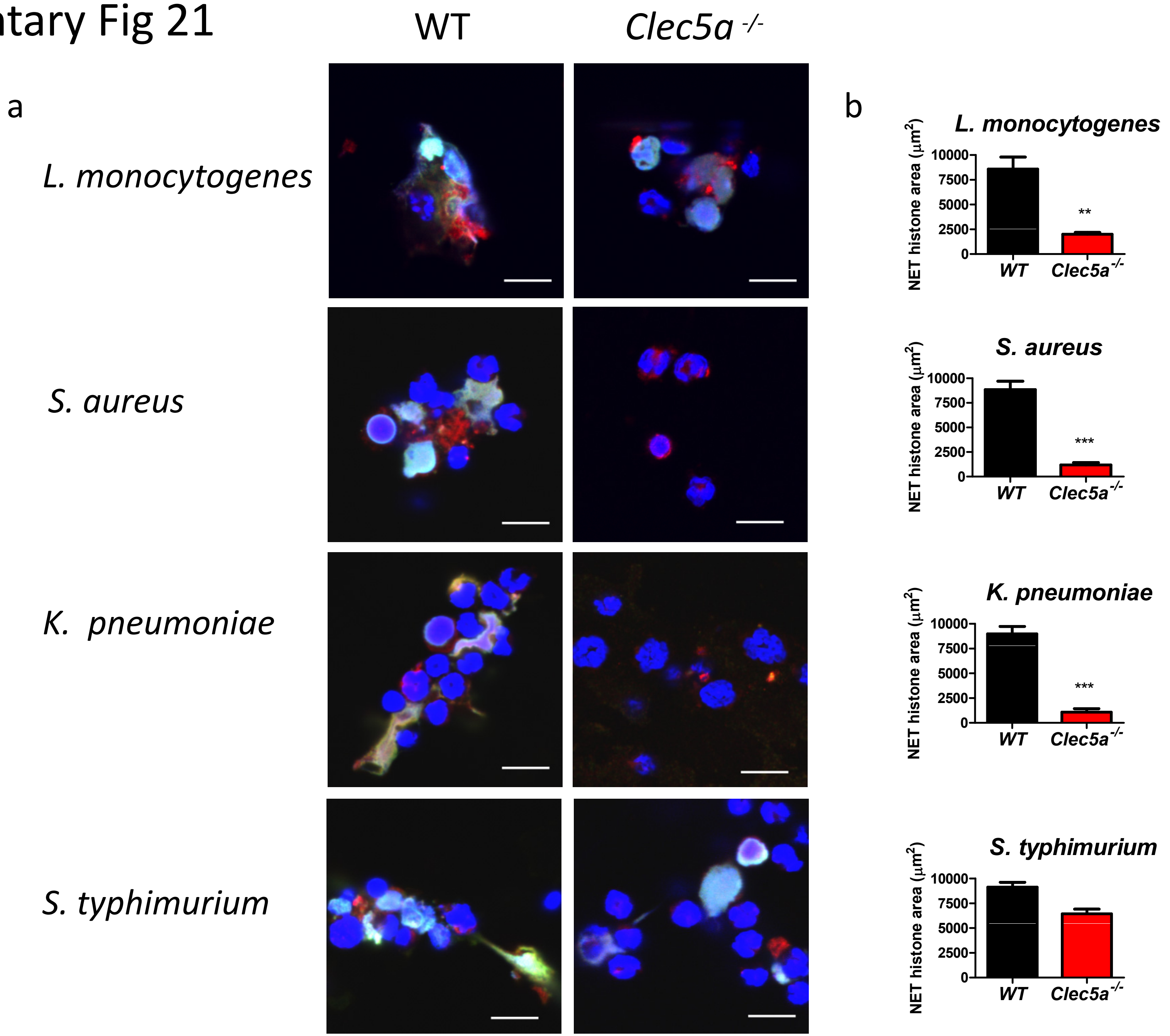

Supplementary Figure 21. CLEC5A is critical for *Staphylococcus aureus* and *Klebsiella pneumoniae*-induced NET formation. (**a**) Mouse neutrophils were incubated with bacteria (MOI 10) for 90 min, fixed and subjected to immunofluorescence staining using Hoechst 33342 (blue), anti-MPO (red) and anti-histone (green) mAbs. Scale bar, 20 µm. (**b**) NET formation was quantitated by determining the histone area (μm^2) per filed. All data were collected and expressed as mean ± s.e.m. from at least three independent experiments. Student's *t* test was performed. $^{**}P<0.01$; $^{***}P<0.001$ for wild type versus CLEC5A knockout cells.

Supplementary Figure 22

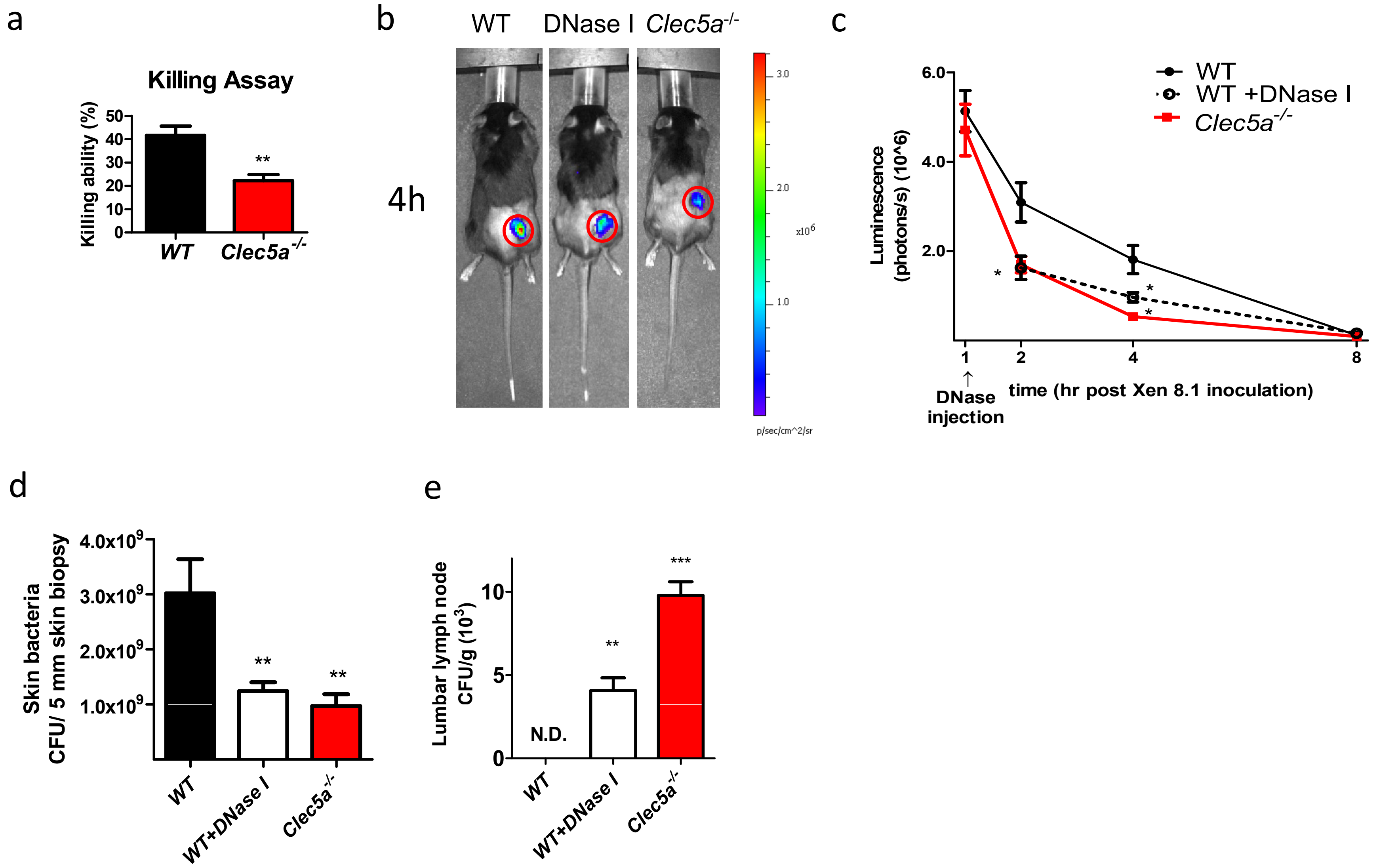

Supplementary Figure 22. CLEC5A is critical to limit *Staphylococcus aureus* dissemination. (**a**) Mouse neutrophils were incubated with live *S. aureus* (MOI 0.1) for 60 min to determine the killing ability. Student's t test was performed. **P<0.01 for wild type versus knockout mice. (**b**) Wild type and *Clec5a*$^{-/-}$ mice were inoculated subcutaneously with live luminescent *S. aureus (*Xen8.1*)* (1$\times$ 10^8 CFUs/mouse). For the WT/DNase-I group, WT mice were inoculated with Xen8.1 and 1 hour later, DNase-I (4 KU) was subcutaneously injected at the site of bacterial infection. Photon image of live luminescent *S. aureus* in the mice skin was detected at 4 h post infection. (**c**) Live luminescent *S. aureus* quantified in the mouse skin was detected at 1, 2, 4 and 8 h post infection (10 mice per group) using the IVIS system. Bacterial load in (**d**) skin biopsy and (**e**) lumbar lymph node were determined at 24 h post infection. Data were collected and expressed as mean $\pm$ s.e.m. from three independent experiments (n=10). One-way ANOVA was performed. *P<0.05, **P<0.01 for WT versus CLEC5A knockout mice.

Supplementary Figure 23

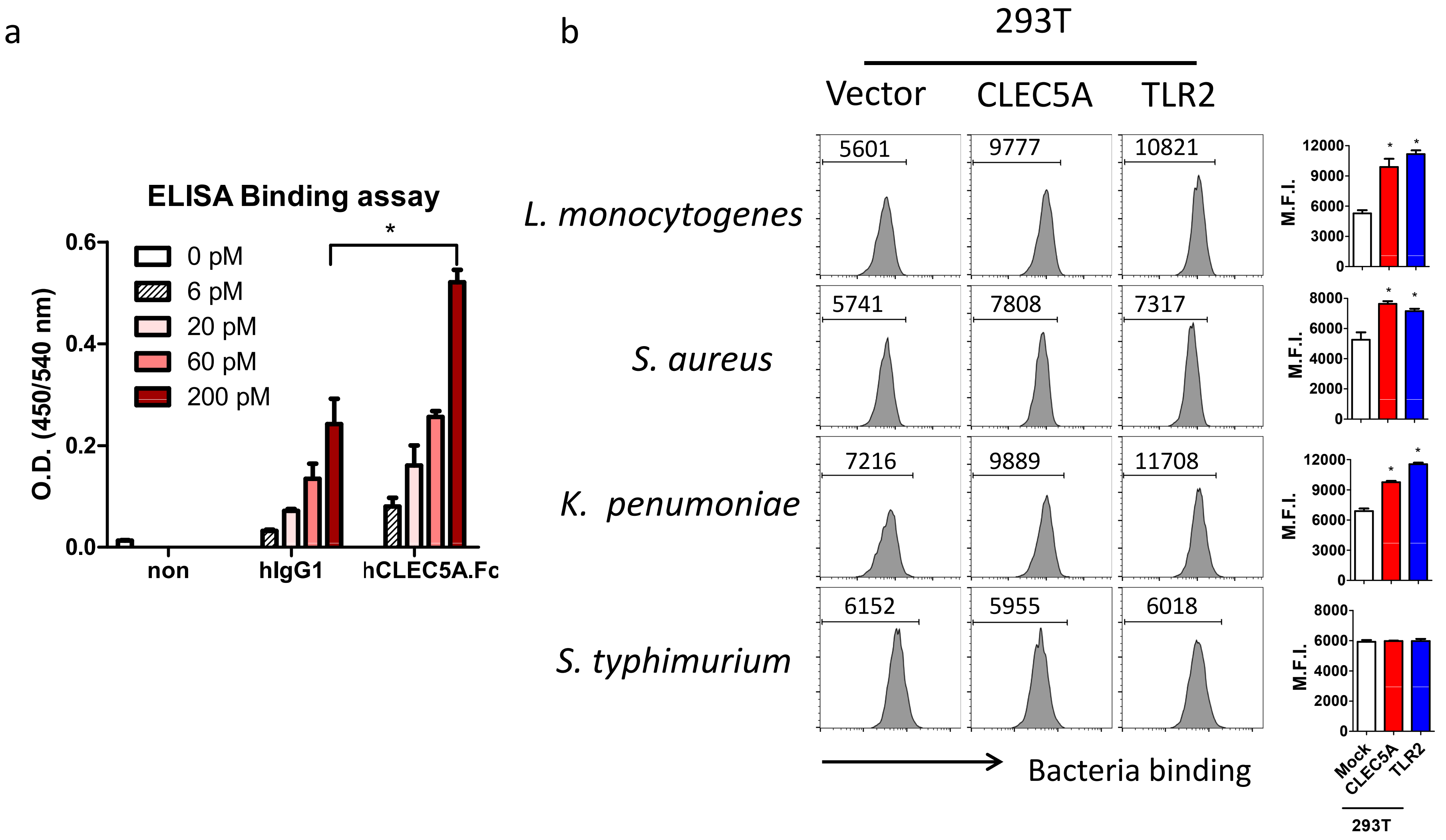

Supplementary Figure 23. Direct interaction of CLEC5A with *L. monocytogenes.* (**a**) Interaction of CLEC5A with *Listeria* cell wall extracts was determined by ELISA. (**b**) Interaction of bacteria (*L. monocytogenes, S. aureus* and *K. pneumoniae*) with 293T cells overexpressing CLEC5A and TLR2, respectively. MFI, mean fluorescence intensity. Data were collected and expressed as mean ± s.e.m. from three independent experiments. Student's *t* test was performed. **P*<0.05 for huIgG1 versus hCLEC5A. Fc fusion protein. One-way ANOVA was performed. **P*<0.05 for CLEC5A-transfected or TLR2-transfected 293T cells versus empty vector-transfected 293T cells.

Supplementary Figure 24

a

b

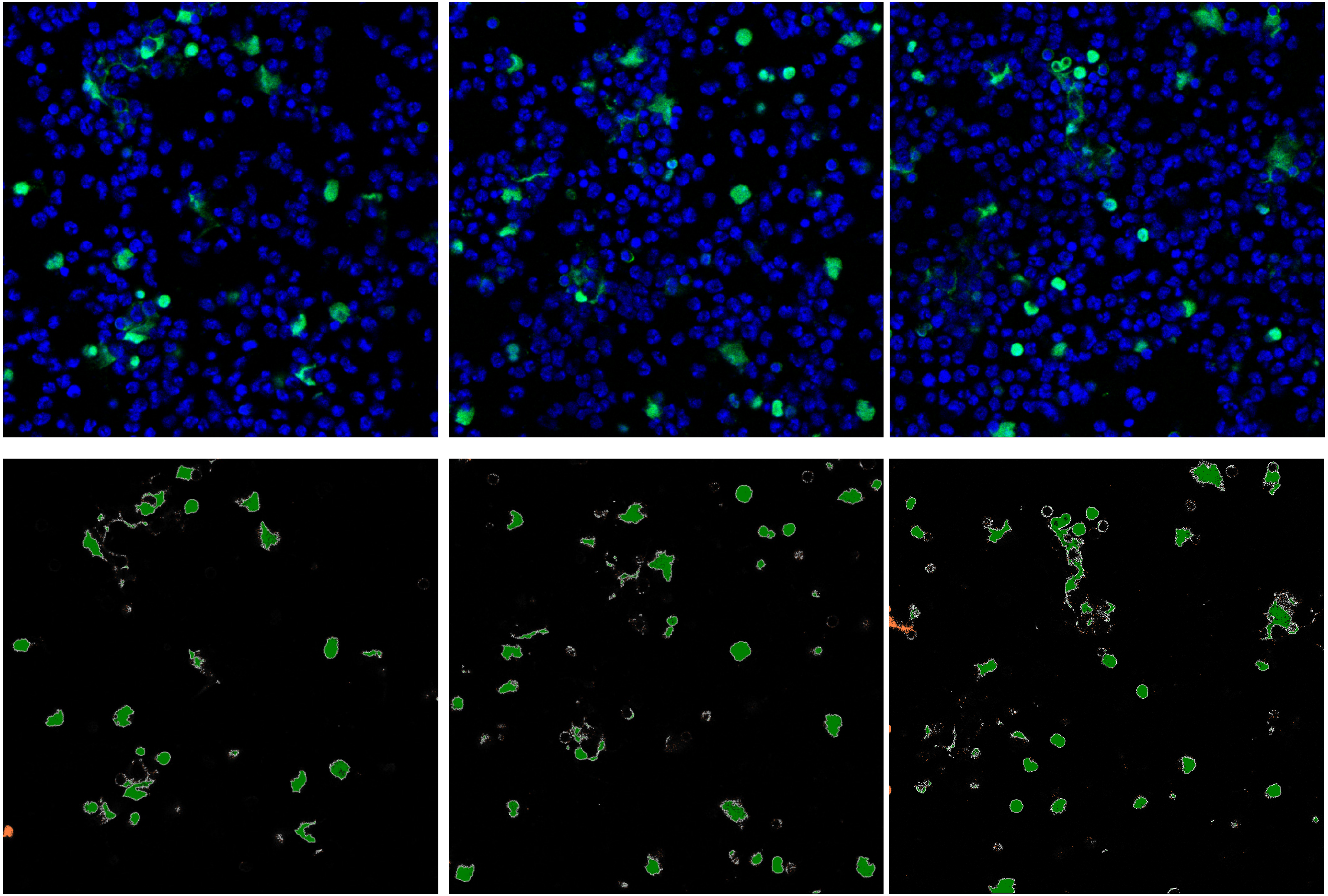

Supplementary Figure 24. Quantification of NET formation. Neutrophils were fixed and incubated with anti-histone H3 antibody (green) and counterstained with Hoechst 33342. (**a**) Images were obtained with confocal microscope. (**b**) Images were processed by MetaMorph® software, thereby the selected areas were counted up to the histone areas.

Supplementary Figure 25

a

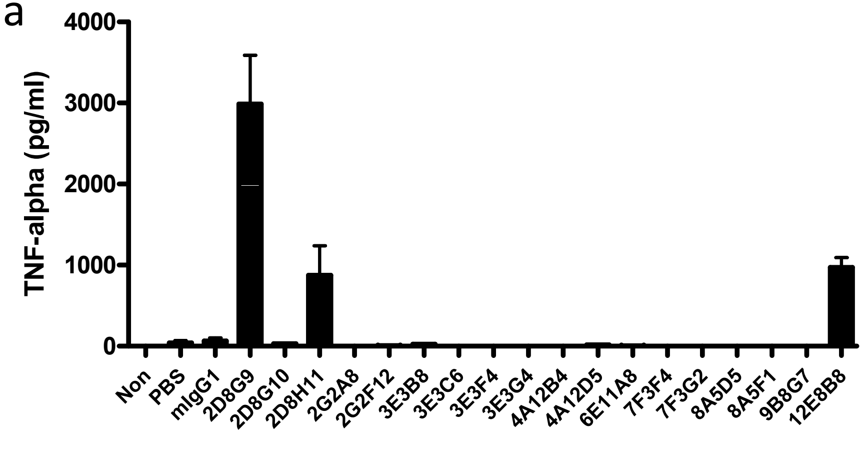

b

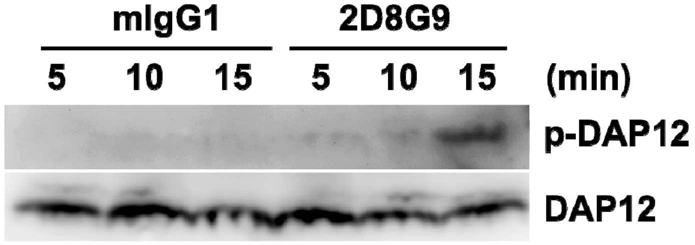

c

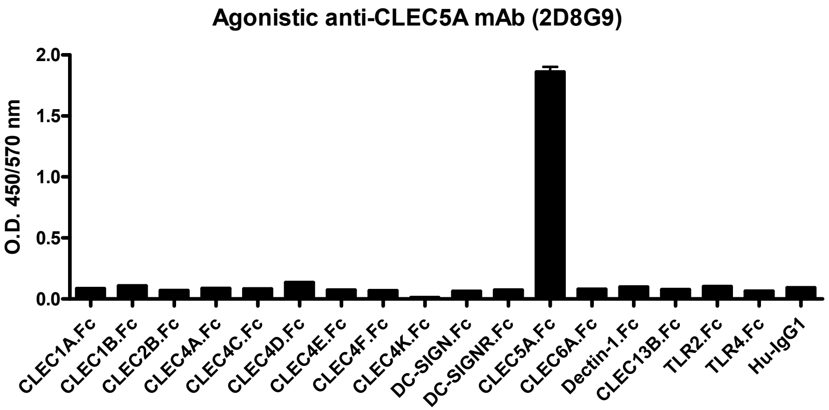

**CLEC5A mAb**	**Recognition Specificity**		**Function**
clone	Human	Mouse	Agonistic
2D8G9	+	+	++
2D8G10	+	+	-
2D8H11	+	+	+
2G2A8	+	+	-
2G2F12	+	+	-
3E3B8	+	+	+
3E3C6	+	+	-
3E3F4	+	±	-
3E3G4	+	+	-
4A12B4	+	+	-
4A12D5	+	+	-
6E11A8	+	+	-
7F3F4	+	±	-
7F3G2	+	+	-
8A5D5	+	±	-
8A5F1	+	±	-
9B8G7	+	+	-
12E8B8	+	+	+

Supplementary Figure 25. Characterization of agonistic anti-CLEC5A mAbs. (**a**) Human primary macrophages were incubated with panel of anti-human CLEC5A mAbs for 30 min, followed by crosslinking with goat anti-mouse IgG antibody to determine their ability to induce TNF secretion. Supernatants were harvested at 24 h post stimulation, and TNF level was measured by ELISA. (**b**) Human primary macrophages (1×10^6) were seeded onto culture plates coated with either isotype Ig control or anti-human CLEC5A mAb (clone: 2D8G9), and cells lysates were harvested at indicated time points to determine the expression of total and phosphorylated DAP12. (**c**) Various human fusion proteins (0.1 µg /well) were immobilized on microtiter plate; anti-CLEC5A mAb (clone 2D8G9, 1 µg/ml) was added, and the binding specificity was determined by ELISA.

Supplementary Figure 26

Fig 1a

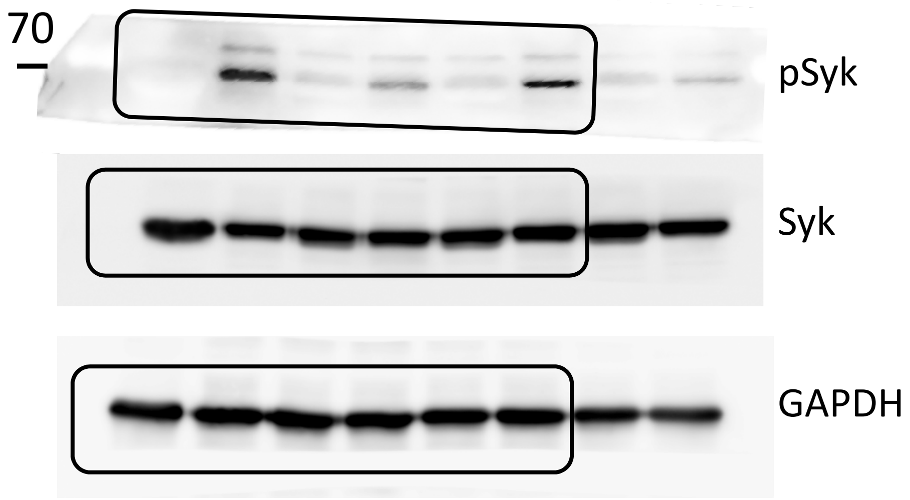

Supplementary Figure 26. Original lumiescence images of the blots presented in the main text . Related to Figure 1a.

Supplementary Figure 27

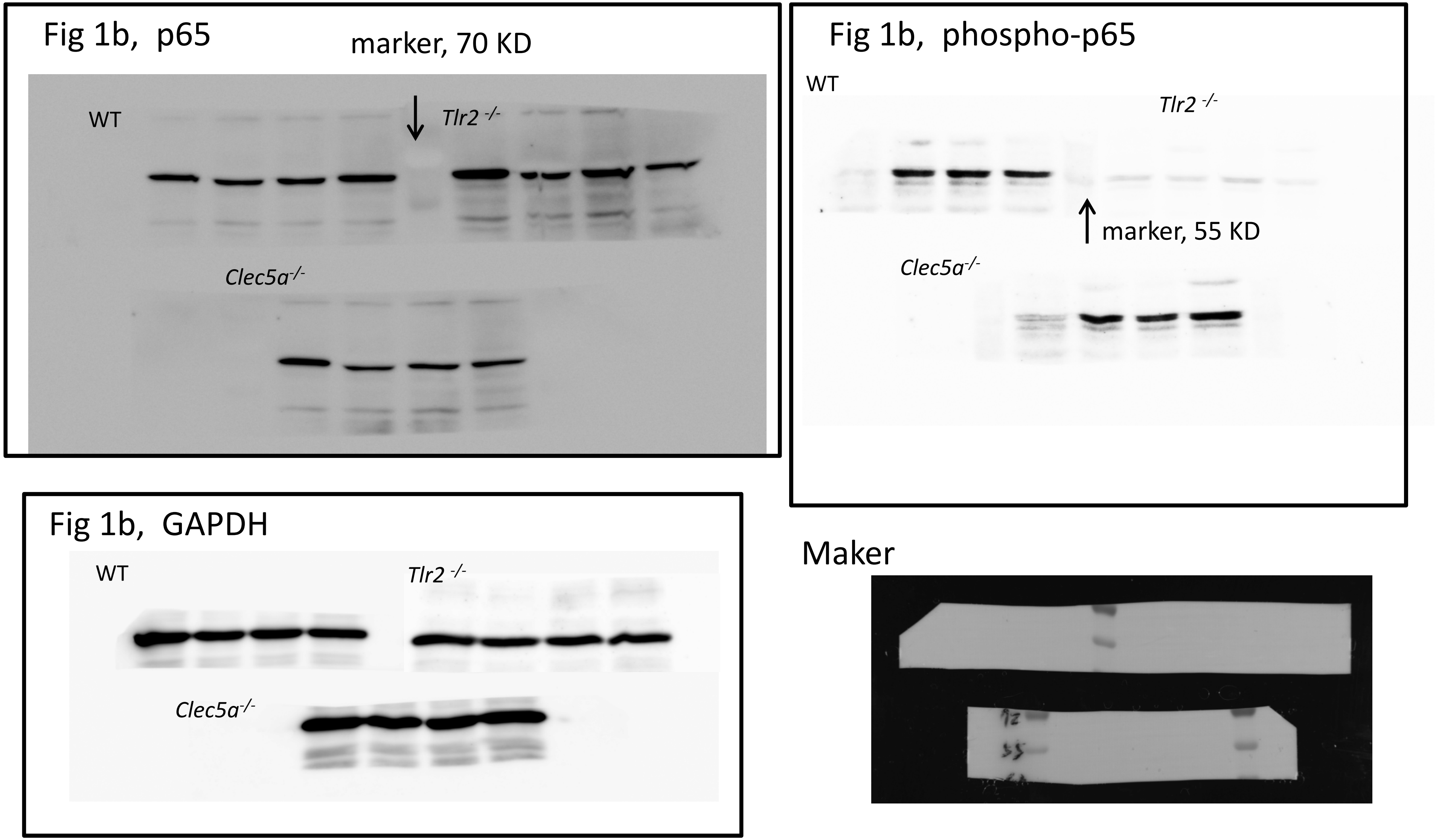

Supplementary Figure 27. Original lumiescence images of the blots presented in the main text . Related to Figure 1b.

Supplementary Figure 28

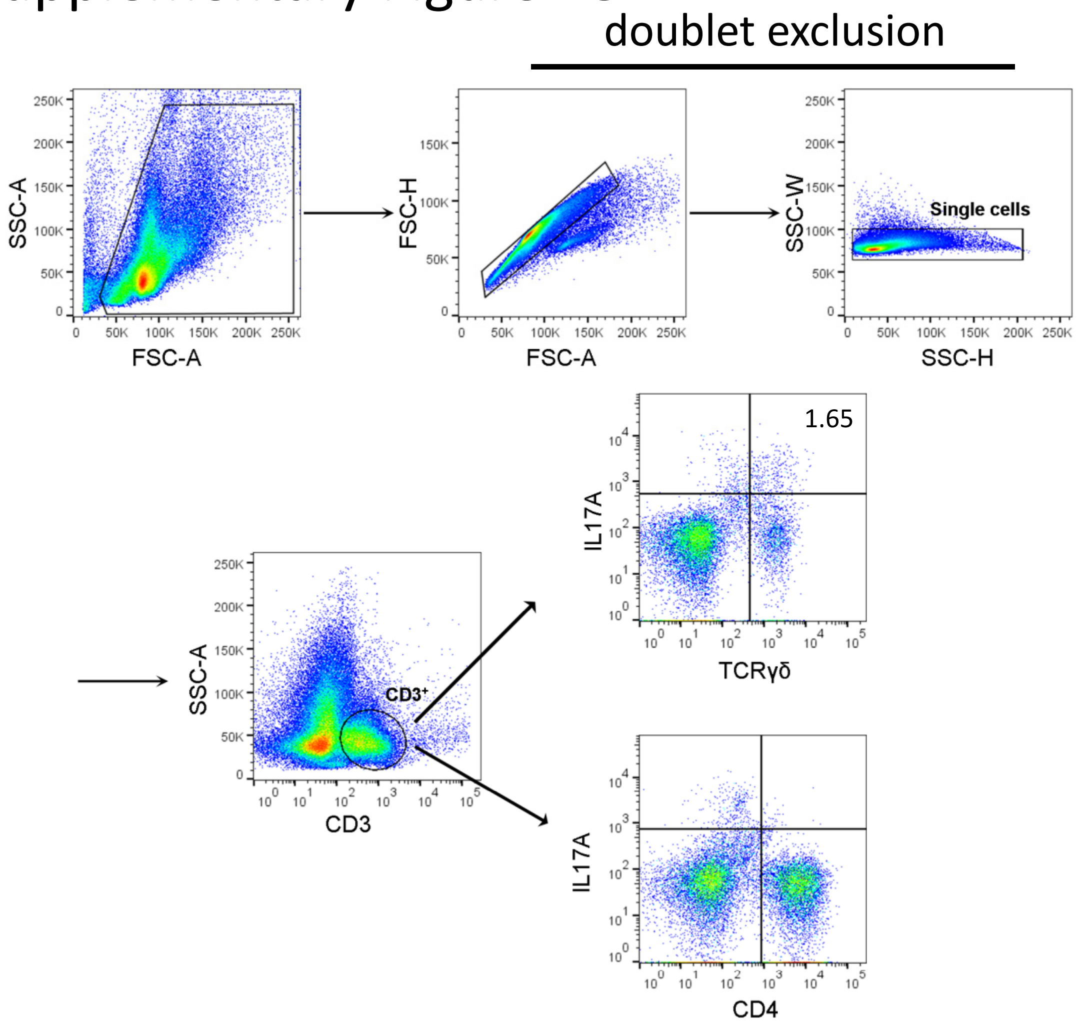

Supplementary Figure 28. Flow cytometry gating schemes. Gating scheme for analyzing IL17A expression in the population of γδ T and CD4^{+} T cells in the mice shown in Figure 4.

Supplementary Figure 29

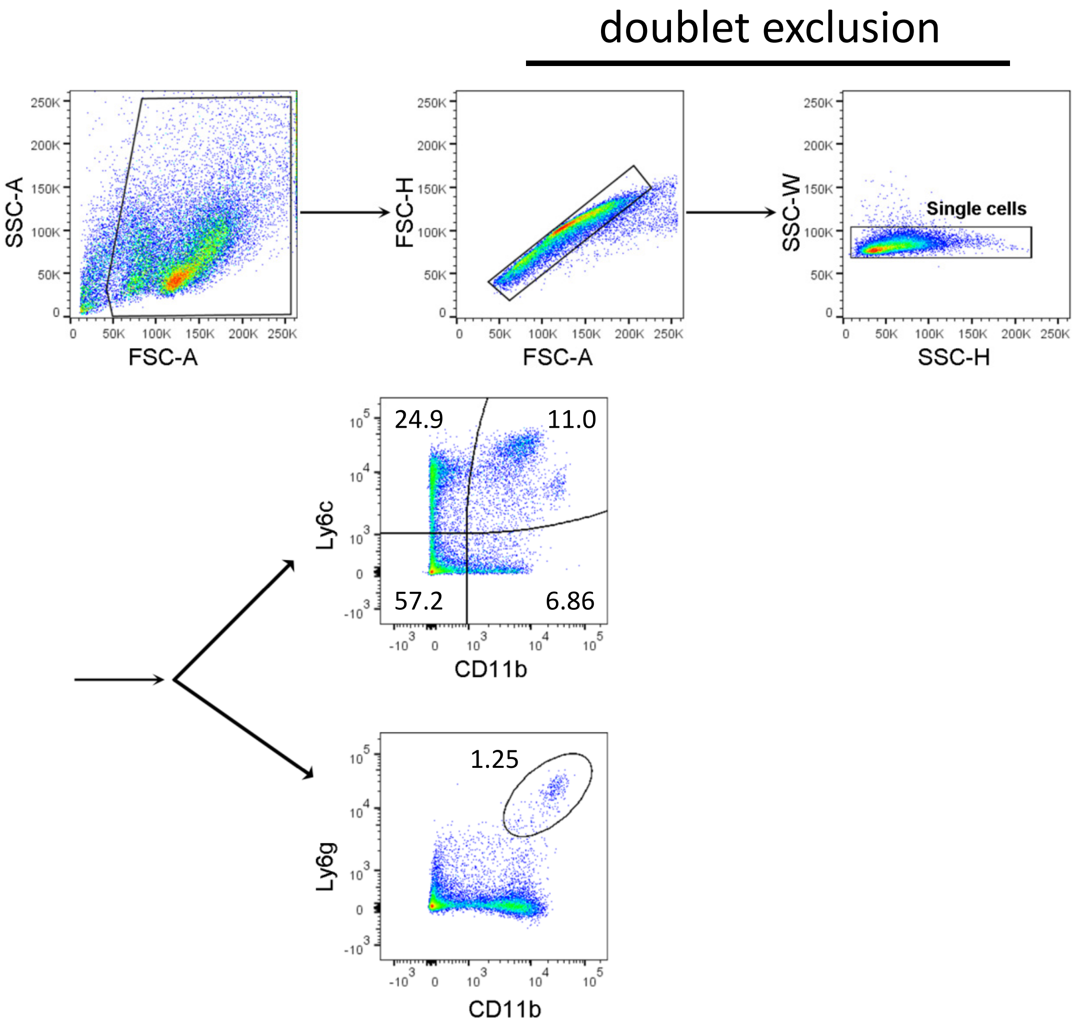

Supplementary Figure 29. Flow cytometry gating schemes. Gating scheme for analyzing the population of $CD11b^{+}Ly6c^{+}$ monocytes and $CD11b^{+}Ly6g^{+}$ neutrophils in the mice shown in Figure 5 and supplementary figure 15 .

Supplementary Figure 30

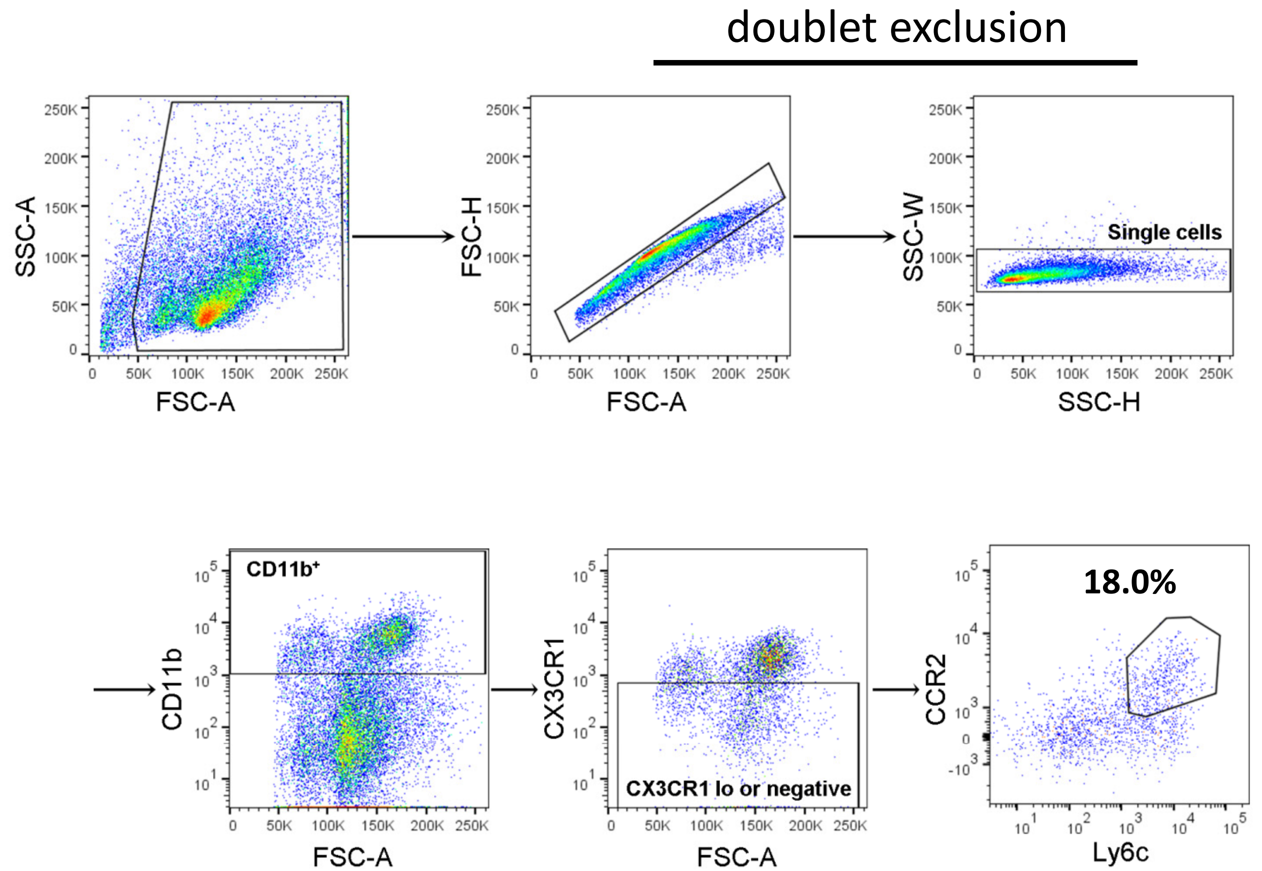

Supplementary Figure 30. Flow cytometry gating schemes. Gating scheme for analyzing the population of inflammatory monocytes (CD11b$^+$CCR2$^+$Ly6c^{hi}CX3CR1^{low}) in the mice shown in supplementary figure 9 and supplementary figure 15.

Supplementary Table 1

Table 1: CLEC5A-microbial glycan interactions*

Chart#	BACTERIA / STRAIN	NAME / STRUCTURE	Average Mean	St DEV	Intensity index	%CV
62	*Pseudomonas aeruginosa O7 7a,7b,7c*	IATS 7,LPS	3439.5	519.3	21.54	15
18	*Escherichia coli K235 LPS*	L2143	3295.3	408.4	20.63	12
282	*Shigella flexneri type 4a*	PS	879.0	90.5	5.50	10
235	*Streptococcus pneumoniae type 17 (Danish type 17F)*	201-X// Capsular PS	548.5	76.2	3.43	14
198	*Shigella boydii type 9*	OPS	544.8	74.2	3.41	14
237	*Streptococcus pneumoniae type 20 (Danish type 20)*	OPS	377	35.7	2.36	9
64	*Pseudomonas aeruginosa O7 7a,7d*	IATS NO, LPS	360.8	50.0	2.26	14
229	*Streptococcus pneumoniae type 4 (Danish type 4)*	173-X// Capsular PS	337.8	52.9	2.11	16
241	*Streptococcus pneumoniae type 34 (Danish type 10A)*	229-X// Capsular PS	335.3	18.6	2.1	6
230	*Streptococcus pneumoniae type 5 (Danish type 5)*	177-X// Capsular PS	324.8	33.6	2.03	10

*CLEC5A-microbial glycan interaction was detected using microbial glycan microarray (MGM), % of CV being less than 20% and Intensity being greater than 2 were chosen in the list. The detail method was described in the section of material and method.

Intensity index: (Avg Mean of sample/Mean of all Avg Mean) Reversed St DEV

%CV: (AvgMeanS-B/StDEV*100%)

Supplementary Table 2

Table 2: Serogroups of *L. monocytogenes*

Name	Serotype	Glycosidic substituents*
10403S	*1/2a*	
EGDe	*1/2a*	
EGDe	*1/2a*	Lack of Rha
EGDe	*1/2a*	Lack of GlcNAc
EDGe	*1/2a*	Lack of GlcNAc and Rha
4MT	*4b*	
XL7	*4b*	Lack of Glc
M44	*4b*	Lack of Gla (+mark reduction of Glc)
19111	*1*	
19112	*2*	
19113	*3*	
19114	*4a*	
19115	*4b*	

Table 2 footnotes:

**GlcNAc: N-acetylglucosamine, Rha: rhamnose, Gal: galactose, Glc:* glucose, *P: phosphate.*
# Schematic structures represent the linear ribitol phosphate repeating units and their glycosidic substituents.

<table>
<tr><th>Serotype</th><th>#Structures of the major WTA types of L. monocytogenes</th></tr>
<tr><td>1/2a</td><td>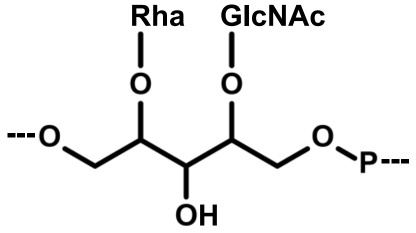
</td></tr>
<tr><td>3</td><td>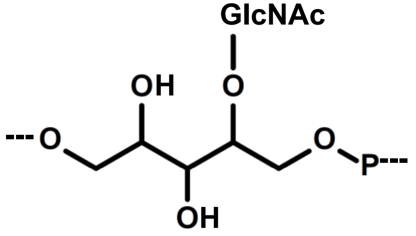
</td></tr>
<tr><td>4a</td><td>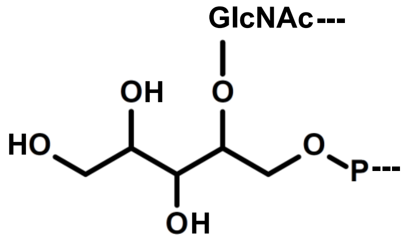
</td></tr>
<tr><td>4b</td><td>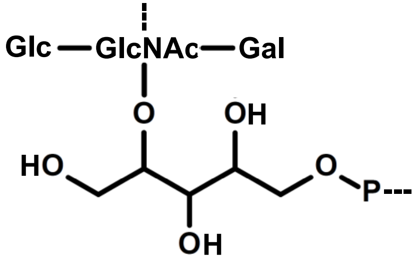
</td></tr>
</table>

Supplementary Table 3

Table 3: mouse Q-PCR primers used in this study

**Gene**	**Forward**	**Reversed**
il-1β	gga gaa cca agc aac gac aaa ata	tgg gga act ctg cag act caa ac
nlrp3	gtg gtg acc ctc tgt gag gt	tct tcc tgg agc gct tct aa
aim2	aca aag tgc gag gaa gga ga	ttt ggc ttt gca gcc tta at
nlrc4	cta cat tga tgc tgc ctt gg	tct ctt cgt ctc tga gtc tc
tnfα	gcc tct tct cat tcc tgc ttg	ctg atg aga ggg agg cca tt
il-17a	gat cag gac gcg caa aca tg	agt ttg ctg aga aac gtg gg
ccl2	gca tcc acg tgt tgg ctc a	ctc cag cct act cat tgg gat ca
ccr2	aga gag ctg cag caa aaa gg	gga aag agg cag ttg caa ag
gadph	gga gga acc tgc caa gta tg	tgg gag ttg ctg ttg aag
